# Place attachment and perception of climate change as a threat in rural and urban areas

**DOI:** 10.1371/journal.pone.0290354

**Published:** 2023-09-06

**Authors:** Thora Tenbrink, Simon Willcock

**Affiliations:** 1 School of Arts, Culture and Language, Bangor University, Bangor, Gwynedd, United Kingdom; 2 School of Natural Sciences, Bangor University, Bangor, Gwynedd, United Kingdom; 3 Net Zero and Resilient Farming, Rothamsted Research, Harpenden, Hertfordshire, United Kingdom; Tokyo Medical and Dental University: Tokyo Ika Shika Daigaku, JAPAN

## Abstract

Climate change is a global threat to ecosystems and the people that depend on them. However, the perceived threat of climate change may vary spatially. Previous research suggests that inhabitants in rural areas show higher levels of place attachment (associating meaning with a specific place) than urbanites, possibly because rural people depend more directly on their local environment. This can shape perceptions and behaviours, such as enhanced willingness to engage in landscape preservation. Here we ask if it also makes rural people perceive climate change as a greater threat, using a representative sample of 1,071 survey respondents from across the United Kingdom (UK) to provide first-order insights. We found that, whilst indicators of place attachment were indeed more frequent in rural areas, the perceived threat of climate change in the most rural locations was lower. We discuss possible explanations for this pattern (including lower levels of awareness of the anthropogenic causes of climate change, lessened first-hand experiences of climate change impacts due to higher levels of regulating ecosystem services, and higher levels of resilience in rural areas related to a closer relationship with nature), and call for further research to investigate this.

## Introduction

Climate change is a global threat to ecosystems and the people that depend on them. However, not everybody perceives this threat to the same degree [1, 2]. For instance, if someone feels highly attached to a place, associating multiple levels of meaning with it, they might also feel more protective of this place (seen for instance in the context of the Great Barrier Reef [3]), and accordingly pay more attention to any changes that might threaten the current state of the place. People living remote from natural spaces and enjoying city life, likely with a high degree of social, digital and recreational aspects that do not depend on a specific location, might feel less affected by climate change, and hence may not perceive a threat to their habitat. As rural people depend more directly on their local environment, this may shape their perceptions and behaviours, such as enhanced willingness to engage in landscape preservation [4]. Here we ask if rural people express a higher degree of place attachment, and perceive climate change as a greater threat, than people living in urban spaces.

Scientific facts leave little doubt that climate change is one of the most pressing global challenges. Atmospheric carbon dioxide concentrations have increased to 417 ppm–the highest in 650,000 years–and, as a result, global temperatures have increased by >1°C since 1880, with the majority of that occurring since 2000 –a period that encompasses 19 of the warmest years on record [5]. Disruptive climate change is already being observed in the UK, with extreme weather events and temperature anomalies becoming increasingly common [6], leading to varied responses among stakeholders [7], and affecting the agricultural system [8]. Between 2011 and 2020, the UK has been, on average, 0.5°C warmer than the 1981–2010 baseline climatic mean and 1.1°C warmer than 1961–1990 [6].

However, the threat of climate change and concomitant disruption vary spatially. For example, some countries, and specific locations within countries, may face higher rates of climate change or be less able to mitigate the negative effects [9]. For example, within environmental social science, it has been argued that in rural areas people and the economy are heavily dependent on their local environment (conceptualised as green-loop systems), whereas in urban areas they are not immediately reliant on local ecosystems for their livelihoods but capitalise on ecosystem service flows from distant ecosystems (conceptualised as red-loop systems) [10]. This relative distance from ecosystems has implications for the socio-economic and cultural valuing of the environment, which can shape perceptions and behaviours [11], potentially including perceptions of the threat of climate change–which, in turn, might impact willingness to undertake climate change mitigation measures [12].

A wide variety of evidence supports red-loop, green-loop theory across a range of ecosystem services, from fuel use [13] to food production [14]. However, there are notable exceptions [15]–for example, proximity to urban green space does not necessarily mean people spend time there [16]. Having potentially closer and more intimate links to nature, are rural residents more aware of climate change and do they perceive it as a greater threat? Are urban inhabitants, residing in a more controlled, man-made environment, maybe protected from some of the impacts by built infrastructure [17], and thus perceive a lower level of threat, possibly none at all in case they do not believe in climate change? In Pakistan, rural residents demonstrated more concern than urbanites [18]; in the UK however, rurality has been observed to be associated with climate skepticism [19]. The location-based difference may be related to different personal experience of climate change [20], reinforcing the importance of understanding people’s perspectives. The answers to these questions are of increasing importance as >55% of the world’s population now live in urban areas and this is predicted to increase to ~68% by 2050 [21].

Valuing one’s environment is frequently linked to the notion of place attachment [22], i.e., the ways in which humans associate meaning with a specific place [23] through a range of facets which can be broadly characterised as anthropocentric (functional and emotive) and geographic (physical and spatial) [24]. Due to the diverse benefits and values that might be appreciated across different types of environment, the subjective experience of place attachment can be affected by multiple factors, complicating any simplistic quantification or comparison across areas [25–27]. Additionally, especially in times of rapid political and societal changes, the factors affecting place attachment do also change [28]. Accordingly, researchers frequently do not agree what exactly constitutes place attachment, which could relate to meanings, emotions, functional/practical considerations, or a sense of identity in relation to a place–all of which relate to different methodological approaches and lead to different predictions. In light of these complexities, the rural and urban distinction can be regarded as one out of several central dimensions that affect satisfaction and place attachment in multiple ways [29].

There is some evidence that rural areas may generally lead to higher levels of attachment [30], possibly because rural people depend on their local environment more [31]. This in turn correlates with a higher degree of willingness to engage in the preservation of the landscape in question [4, 32], linked to rural community resilience [33]. With climate change affecting local communities, individuals either need to adapt or to migrate. As higher place attachment implies a higher motivation for staying in a place and adapting, rather than giving up a place associated with culture and identity [34], the link between place attachment and climate change becomes particularly crucial for policy responses [35, 36].

Although numerous local place studies in different areas are available, to our knowledge no nation-wide representative study so far has attempted to capture degree of place attachment relative to degree of rurality, or systematically explored the link to perceived threat of climate change. Indeed, degree of place attachment as such is a slippery concept due to the multiple factors relevant in a context. If asked directly to grade one’s attachment on a scale, responses will inevitably depend on the respondents’ individual understanding of attachment in regard to any of the factors identified in the literature as meaningful place facets [24]. Accordingly, most research in the area avoids adopting simple, uni-dimensional Likert scales to measure place attachment [37], and generalised place attachment scores have been calculated on the basis of scenario factors in various ways (e.g., [38–40]).

Nevertheless, the problem persists that Likert-scale based survey questions inevitably restrict people’s responses to the types of pre-formulated sentiments given to them, thereby affecting the range of possible answers, and possibly biasing them by the mere mention of a sentiment. Wording and design of the survey is decisive, with even small changes potentially affecting the results. These concerns have led to an attested move towards more qualitative or free-text based methods [41]. Apart from quantitatively measuring levels of importance of various place facets, crucial aspects of place attachment can be derived from the discourse about places, i.e. the way people talk about and represent places in language. Indeed, most research on place ultimately relies on language, through survey questions, interviews or simply considerations of what it means to be attached to places. Abundant research demonstrates the benefits of using linguistic discourse analysis to identify concepts represented in unconstrained natural language data [42, 43]. In the spatial domain, insights gained in this way include culture-dependent ways of conceptualising object configurations [44, 45], key features of route concepts [46], and profession-based differences in understanding spaces [47]. However, the linguistic expression of place attachment requires further scrutiny [48].

Here, we systematically examine free-text responses on place attachment in relation to the perceived threat of climate change across the rural-urban spectrum in the UK, as perceived by its inhabitants: in modern times of mobility and remote working, participants’ perceptions may depart from objective measures such as number of inhabitants, which also overlook the particular location that a person inhabits. Specifically, we evaluate the relationship between self-determined rurality and both place attachment and the perceived threat of climate change, statistically controlling for the possible effects of gender, socioeconomic group, region and age. Based on red-loop, green-loop theory [10], we hypothesise that as the perception of rurality increases, so do place attachment as reflected in free-text responses and the perceived threat of climate change.

## Materials and methods

Our study addresses the relation between place attachment (sense of place) and climate change, with particular focus on the influence of location, i.e. whether the threat of climate change to sense of place is felt more in areas that inhabitants perceive as rural, than in areas that they perceive as urban. Using a fast-track but tightly constrained data collection approach that enabled us to efficiently reach a nationally representative sample without imposing on participants’ privacy, we designed a survey to ask the following three questions:

1. On a scale of 1–5, where 1 is ‘very rural’ and 5 is ‘very urban’, how rural or urban do you live?
2. On a scale of 1–5, where 1 is ‘not a threat at all’ and 5 is ‘extremely threatening’, how big a threat is climate change to your local area in the foreseeable future?
3. Please describe your local area, in terms of what it means to you personally.

Question 1 operationalised the potentially tricky dichotomy between urban and rural areas by focussing on the participants’ subjective perception. For current purposes, objective facts, such as number of inhabitants in a village or town, are less important than the degree to which inhabitants themselves feel that they live in a rural or urban area. Such an approach minimises collection of personal data about respondents, but rules out prescribing respondents levels of rurality (e.g. via extraction of land cover data derived from postcode locations). A Likert scale is appropriate to capture these subjective perceptions. It can also be expected that participants are sufficiently familiar with the notion of climate change threat to be able to place their own perception on a Likert scale (Question 2).

In contrast, place attachment cannot reliably be elicited through a single Likert scale, as this notion encompasses multiple facets [24]. Hence, Question 3 elicited free-text responses that allowed us to identify and systematically extract key facets mentioned by participants as indicators for place attachment, inspired from relevant literature on place attachment indicating the importance of various factors such as people (friends, family, neighbours) in addition to spatial and descriptive aspects, and associated facts and functions (village, sea front, heritage), plus different degrees of emotional appreciation that are frequently reflected in praising adjectives (lovely, fantastic, pleasant).

Anonymous, fast-track data collection was conducted using the online survey distribution platform FindOutNow (https://findoutnow.co.uk/), with the aim of efficiently obtaining a nationally representative sample of ~1,000 respondents. FindOutNow specialises in ‘micro-surveys’, limiting surveys to a maximum of three questions at a time to ensure a timely yet considered response (https://findoutnow.co.uk/services). FindOutNow distributed the survey through Pick My Postcode, a free, online postcode lottery whereby ‘bonus’ credit can be earned by completion of surveys, but only redeemed on winning the postcode lottery (https://pickmypostcode.com/). Through the mechanisms of the Pick My Postcode platform, there is no room for lengthy ethical statements for each individual survey, but the platform has a site for survey terms and conditions which explicitly states that *“Participating in any research is entirely up to you and is not required’* (https://help.pickmypostcode.com/article/103-third-party-surveys). Furthermore, Pick My Postcode’s *“services and the Website are for use by members 18 years old and over only*. *[They] do not market to users under the age of 18 and do not intentionally collect data on users under the age of 18”*. Through these services, the survey was completed by >1,000 people in June 2021, from which a nationally representative sample of ~1,000 was drawn randomly from the responses (stratified by gender, socioeconomic group, region and age). This is not an entirely random sample as respondents are all members of Pick My Postcode. However, as this is likely unrelated to the research goals, the effect is similar to a random sample. As the data were collected anonymously through the online survey platform and the questions were not personal, the authors did not have access to information that could identify individual participants during or after data collection. Ethics approval was obtained through Bangor University (05/05/2021 TT1).

The free-text data were analysed as follows. Wordcount is a simple quantitative measure that potentially indicates individual verbosity, engagement level with this survey, and strength of emotion and extent of relevant thoughts that come to mind. While individual verbosity is a random factor that adds noise, strength of emotion and extent of relevant thoughts may affect engagement level; hence word count might be a first indicator of the strength of place attachment.

Furthermore, the free-text responses contained key words and phrases that were recognisable as indicators for place attachment to different degrees. For current purposes these were identified as search terms to allow for a semi-automatic approach to coding, and then categorised into a gradual scale ranging from weak to strong indicators of place attachment. Categorisation was based on examination of the data, inspired by the literature on place facets as reported above [24], and captured by an overarching definition as follows (the word lists are mutually exclusive and complete; i.e., all search terms counted are listed):

- **Weak indicators of place attachment** were primarily descriptive rather than evaluative, or people- rather than place-oriented, generic, and factual, namely: *close*, *place to live*, *friends/friendly*, *community*, *comfortable*, *neighbors/neighbourhood*, *convenience/t*, *ok(ay)*, *alright*, *park*, *quiet*, *clean*, *tidy*, *rural*, *familiar/y*, *safe*, *village*, *countryside*, *valley*, *suitable*, *seafront*, *coastal*, *suburban*, *open*, *forest*, *garden*, *cool*, *easy*, *fine*, *decent*, *social/able*, *quaint*, *small*, *tree*, *mountain*, *beach*.
- **Medium indicators of place attachment** conveyed positive evaluation without enthusiasm, fairly generic rather than clearly tied to a place: *great*, *nice*, *pretty*, *character*, *nature*, *picturesque*, *peace*, *leafy*, *unspoilt*, *scenic*, *relaxing*, *good*, *freedom*, *wild(life)*, *happy*, *pleasant*, *welcoming*, *lot*, *like*, *green*, *tranquil*, *interesting*, *calm*, *scenery*.
- **Relatively strong indicators of place attachment** were clearly place oriented and positive but not highly emotional: *home*, *beauty/iful*, *special*, *lovely*, *views*, *idyllic*, *oasis*, *unique*, *plenty*, *memory/ies*, *best*, *grew up*, *root(s)*, *important*.
- **Very strong place attachment** was conveyed in our data by: *love*, *refuge*, *fantastic*, *wonderful*, *lucky*, *incredible*, *heritage*, *quintessential*, *sanctuary*, *wouldn’t (want to live anywhere else)*, *awesome*, *ideal*, *precious*, *perfect*, *bliss*, *amazing*.

The listed search terms were identified *post-hoc* on the basis of the collected data, following methodological considerations motivated and detailed in [43] and related work reported in [47, 49, 50]; see also [51, 52] for a similar approach in different fields. In this instance, a *post-hoc* approach was necessary because of the absence of a clearly established catalogue of linguistic indicators of place attachment, along with the flexibility and scenario dependency of each linguistic data set. Decisions about inclusion and weighting of each lexical item were based on careful examination of the contexts in which the item occurred, aiming to capture underlying concepts and associations to the extent possible (for transparency, refer to S1 Table to access the full original data set). While the grading of strength of attachment primarily captures intensity of associated affect and emotion [53], the range of identified search terms also reflects other key facets of place attachment identified in the literature, such as social relations, spatial location, environmental qualities, activities, affordances and experiences, history and culture [24].

It is important to note that inevitably, not every linguistic expression will have been captured in this way. It is, for instance, possible that an utterance contains very few or none of the above search terms, yet can be read as indicating place attachment in its own way. However, there is a strong advantage of a clearly defined, entirely transparent and reproducible operationalisation of this kind: it reduces the high degree of ad-hoc intuition and subjectivity associated with the alternative solution available for current purposes, namely manual coding of each free-text response on a scale of place attachment [43]. Both methods are widely adopted in linguistic discourse analysis [54].

Once identified, the search terms were automatically counted using Excel formulas, leaving no room for human error in this analysis step except for spelling errors in the input data; these were corrected wherever spotted. Additionally, a few adjustments needed to be made manually to avoid misleading counts. For instance, the expression ‘lot’ was included for the high frequency of answers that simply said ‘a lot’—i.e., it means a lot to me—and those that referred to ‘lots’ of nice things. To avoid automatically counting cases that were identified as not fitting this interpretation, a ‘%’ sign was inserted in the input data, e.g. ‘not a l%ot’. This part of the procedure implies a margin for error.

To identify a simple quantitative score for purposes of further analysis of relations to other factors in the data set, the four categories were weighted and added up to a ‘place attachment score’ following Eq 1:

Placeattachmentscore=(countofweakindicators*1)+(countofmediumindicators*2)+(countofrelativelystrongindicators*3)+(countofstrongindicators*4) (Eq 1)

Hence, search terms expressing higher degrees of place attachment weighed more. However, note that the length of the search term lists above decreases, with more expressions identified as weak than strong indicators (with the other categories ranging between those extremes). Therefore, an utterance that praises various local aspects in moderate terms (such as ‘most famous village in the country, a beautiful large village with lots of history’–score: 8) will often receive a higher place attachment score than an utterance that simply says ‘I love it’ (score: 4). This ties in with the above observation that number of words alone may, to some extent, reflect degree of attachment. For this reason, it is also appropriate to score in absolute numbers rather than relative frequencies.

The data were analysed using descriptive statistics and ordinal logistic regression. Descriptive statistics were used to determine the overall proportion of the sample’s self-determined location on the rural-urban spectrum, as well as level of threat of climate change. Both self-determined rurality and perceived threat of climate change were categorised as ordinal variables, from ‘very rural’ to ‘very urban’, and from ‘not a threat’ to ‘extremely threatening’ respectively. Using the polr function (MASS package v 7.3–51.6), we conducted a ordinal logistic regression model comparing the perceived threat of climate change with both rurality and place attachment score (Eq 2).


Threat=Rurality+Gender+SEG+Region+Age+PlaceAttachmentScore+Wordcount (Eq 2)

Where:

- Threat is an ordinal variable on a scale of 1–5, where 1 is ‘not a threat at all’ and 5 is ‘extremely threatening’
- Rurality is an ordinal variable on a scale of 1–5, where 1 is ‘very rural’ and 5 is ‘very urban’
- SEG is a nominal variable describing social-economic group using the NRS social grades (whereby AB represents higher & intermediate managerial, administrative, professional occupations, C1 represents supervisory, clerical & junior managerial, administrative, professional occupations, C2 represents skilled manual occupations, and DE represents semi-skilled & unskilled manual occupations, unemployed and lowest grade occupations).
- Age is a nominal variable grouped in decades between 18–29 and >80 years
- Place Attachment Score is a continuous variable with higher values indicating higher degree of attachment
- Wordcount is a continuous variable.

To investigate the links between place attachment and rurality, we performed a general linear model using the same explanatory variables (Eq 3):

PlaceAttachmentScore=Rurality+Gender+SEG+Region+Age+Wordcount (Eq 3)

All analyses were performed using R Statistics version 4.0.2.

## Results

Our survey distribution resulted in a nationally representative sample of 1,071 respondents (S1 Table). The respondents were approximately equally divided across gender (526 men [49.1%] and 545 women [50.9%]), age categories (18–29: 17.4%, 30–39: 19.0%, 40–49: 18.4%, 50–59: 15.7%, 60–69: 17.2%, 70–79: 10.1%, >80: 2.1%), socioeconomic group (AB: 27.0%, C1: 28.0%, C2: 20.0%, DE: 25.0%), and region (East Midlands: 7.4%, East of England: 9.5%, Greater London: 12.8%, North East: 13.4%, North West: 11.4%, Scotland: 8.7%, The South East: 13.9%, The South West: 8.8%, Wales: 5.1%, West Midlands: 9.1%; Table 1). Overall, 5.8% of respondents scored themselves as ‘1 –very rural’ and 16.6% as ‘5 –very urban’, with 17.8%, 36.6% and 23.9% as 2 to 4 respectively. Overall, 9.3% rated climate change as ‘1 –not a threat at all’ and 13.6% as ‘5 –extremely threatening’, with 16.9%, 40.2% and 19.9% as 2 to 4 respectively.

**Table 1:** The distribution of our nationally representative sample (n = 1,071).

Characteristic	Percentage (%)	Quota (lower to upper)	Sample obtained
**Gender**			
Male	49.1	500–552	526
Female	50.9	518–572	545
**Age Range**			
18–24	12.0	122–135	128
25–34	17.0	173–191	181
35–44	17.7	180–199	188
45–54	17.6	179–198	189
55–64	14.9	152–168	159
65+	20.9	213–235	226
**Socioeconomic group**			
AB	27.0	275–304	269
C1	28.0	285–315	300
C2	20.0	203–225	214
DE	25.0	254–281	268
**Region**			
East of England (Eng)	9.6	97–107	102
East Midlands (Eng)	7.5	76–84	79
Greater London (Eng)	12.8	130–143	137
North East (Eng)	13.2	135–149	143
North West (Eng)	11.5	117–129	122
South East (Eng)	13.9	142–157	149
South West (Eng)	8.8	89–98	94
West Midlands (Eng)	9.1	92–102	97
Wales	5.1	52–58	55
Scotland	8.7	88–98	93

Where AB represents higher & intermediate managerial, administrative, professional occupations, C1 represents supervisory, clerical & junior managerial, administrative, professional occupations, C2 represents skilled manual occupations, and DE represents semi-skilled & unskilled manual occupations, unemployed and lowest grade occupations. England is represented by Eng. See S1 Table for the raw data.

The ordinal logistic regression (Eq 2) showed self-determined rurality to be the only significant predictor of the perceived threat of climate change (S1 Fig). As rurality is an ordinal variable, the model fits a series of polynomial functions to the levels of the variable: the first is linear (.L), the second is quadratic (.Q), the third is cubic (.C), and the last (^4) is to the power four (S2 Table). Thus, the reporting statistics for rurality in Eq 2 are t = 4.57, -2.18, 1.70, and -1.55, and p < 0.001, 0.05, 0.1, and 0.13 respectively (all other variables: p > 0.05; S2 Table). Similarly, the general linear model (Eq 3; Adjusted R-squared: 0.2492; F-statistic: 15.8 on 24 and 1046 df, p-value: < 0.001) showed significant differences in place attachment scores related to both rurality (t = -2.86, -1.09, 0.61, and 0.28, and p < 0.001, 0.01, 0.3, and 0.6 respectively) and word count (t = 17.00, p < 0.001), with p-values for all other variables > 0.05 (S3 Table). Thus, we reran both models only retaining statistically significant explanatory variables (i.e. comparing rurality with perceived threat, and place attachment score to both rurality and wordcount; Eqs 4 and 5).


Threat=Rurality (Eq 4)

PlaceAttachmentScore=Rurality+Wordcount (Eq 5)

**Fig 1:**
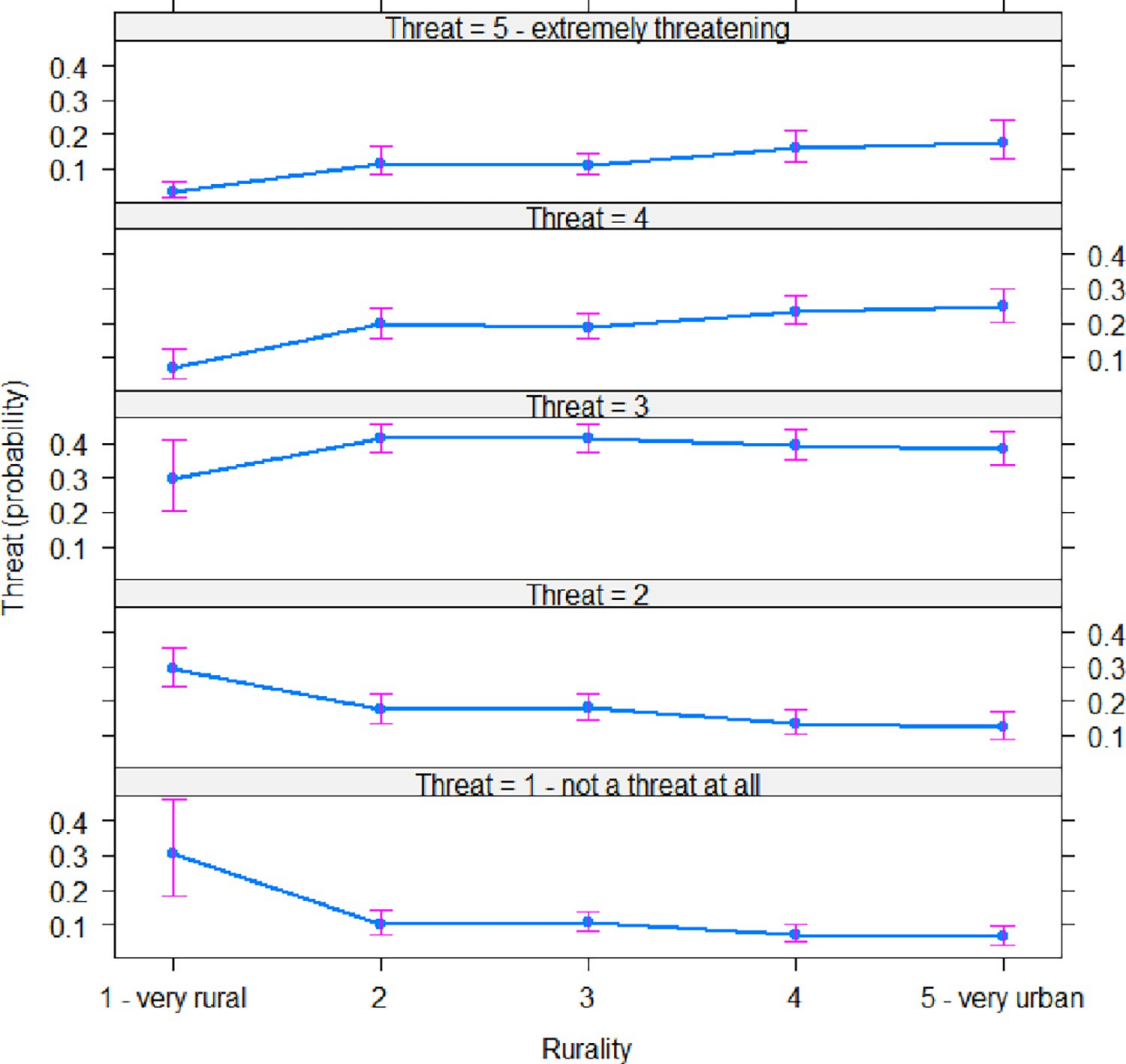
An effects plot comparing self-defined rurality with the perceived threat of climate change (n = 1,071; Eq 4). Rurality was a significant predictor of perceived threat of climate change, with the threat of climate change perceived by those self-describing as ‘very rural’ lower than all other rurality categories (S2 Table).

**Fig 2:**
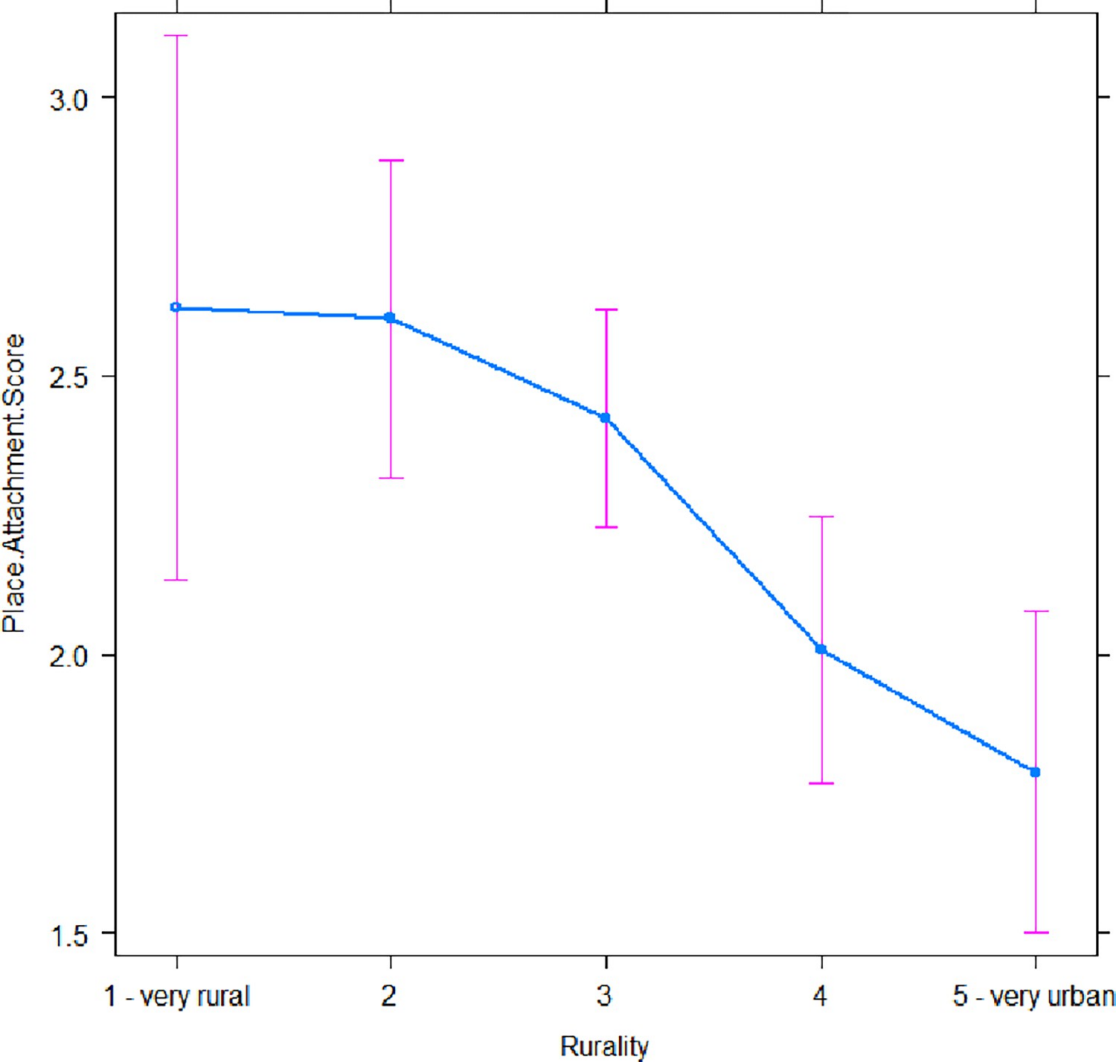
An effects plot comparing self-defined rurality with the place attachment score (n = 1,071; Eq 5). Rurality was a significant predictor of place attachment score, with the most rural people scoring highest (S3 Table).

Here, we report results from the simplified models (Eqs 4 and 5), but results from the full models (Eq1 and 2) are consistent (S2–S5 Tables). As described above, rurality was a significant predictor of perceived threat of climate change (t = 5.05, -2.44, 1.94, and -2.23, and p < 0.001, 0.05, 0.06, and 0.05 respectively), with the threat of climate change perceived by those self-describing as ‘very rural’ lower than all other rurality categories (Fig 1; S4 Table). Indeed, the probability of a ‘very rural’ respondent reporting climate change as ‘1 –not a threat at all’ was ~3-fold higher than all other categories on the rural-urban spectrum (Fig 1). The general linear model (Adjusted R-squared: 0.2424, F-statistic: 69.47 on 5 and 1065 DF, p-value: < 0.001) showed that free text descriptions with higher word count obtained a higher place attachment score (with each additional word raising the score by 0.15; t = 17.87, p < 0.001; S2 Fig), and the most rural people had the highest place attachment scores (t = -3.72, -1.00, 0.75, and 0.51, and p < 0.001, 0.4, 0.5, and 0.7 respectively; Adjusted R-sq: 0.2429; Fig 2; S5 Table).

Finally, we note that about 1/5 of respondents (276 out of 1,071; 48 rural or very rural, 153 urban or very urban) scored 0 for place attachment–i.e., they did not use a single word that would have indicated place attachment according to the criteria used for current purposes. Responses frequently indicate a degree of indifference, as in ‘I live here’, ‘not much’, ‘ok town’, or more elaborately: ‘it doesn’t mean a lot to me as I don’t see myself here long term’.

## Discussion

We conducted a nationally representative study across the UK to address relationships between self-assessed degree of rurality, place attachment as reflected in free-text responses, and perceived climate change threat. Our results demonstrate the utility and feasibility of a formula based on linguistic indicators to derive a place attachment score from free-text responses, taking into account various types of contributors to place attachment as well as intensity, and keeping coding subjectivity to a minimum due to using a transparent search term based procedure. They also confirm that rurality is a major factor consistently affecting the degree to which people feel attached to their local area across the UK, with highest attachment expressed in the most rural locations, and lack of explicit place attachment associated with the most urban locations (55.4% of those who scored zero for place attachment perceived their location as urban or very urban). The latter may be related to a sense of ‘placelessness’, with no perceived need to relate to a place [55]. This phenomenon might be increasingly prominent in a globalised world characterised by high mobility [56], affecting the ways in which people bond with places [57], making the current home location possibly less important than other places that are, or have been, relevant to people’s lives. Altogether, these findings correspond to previous, more localised findings (e.g., [30, 31]), including that people who live closer to nature may generally feel more connected to it [10]–the many factors contributing to place attachment notwithstanding [24, 28].

However, contrary to our predictions, the greater place attachment found in rural areas is not associated with greater perceived threats from climate change. Instead, our results suggest that, whilst the perceived threat of climate change does vary across much of the rural-urban spectrum, lower threats are perceived in the most rural locations. Research on the impacts of climate change across the rural-urban spectrum is rare (e.g. there is significant neglect of urban issues in climate change–conflict literature, with the bulk of research directed to the rural areas, and cities receiving limited study [58]). As such, the drivers of this pattern require much further study, however, in the remainder of the discussion we propose three possible reasons for the lower levels of perceived threat of climate change in rural areas, despite higher levels of place attachment.

One possible explanation is that this pattern may be driven by climate change awareness. For example, using 2007–2008 data from 119 countries (representing over 90% of the world’s population) [59], Lee *et al*. [60] identified that rurality was a key predictor of climate change awareness in China–with poorly educated, lower-income residents living in a rural area being the least aware of climate change, whereas those who are highly educated and urban are the most aware [60]. However, rurality was a less important predictor of climate change awareness on a global scale, with educational attainment the single strongest predictor of climate change awareness, and understanding the anthropogenic cause of climate change the strongest predictor of climate change risk perceptions, particularly in Latin America and Europe [60]. Given strong correlation between SEG and education level [61], and high awareness of the anthropogenic causes of climate change [62, 63] in the UK, it is perhaps unlikely that this is driving our results. Nevertheless, there may be a possible influence of political differences across the urban-rural gradient; it is well documented that rural inhabitants tend to have more conservative views [64], which in turn can affect how climate change impacts are interpreted [65].

An alternative explanation can be derived from the ecosystem services concept. Whilst we predicted that rural residents are more directly connected to nature [10] and so maybe perceived a greater threat of climate change than urban inhabitants, these strong, direct connections to nature might actually *reduce* their lived experience of climate change and, concomitantly, their perceived threat [20]. By their nature, rural areas have higher levels of greenspace than urban areas and, as a result, often have higher levels of regulating services that act to lessen the impact of extreme events on local people [15]. This might explain the higher degree of climate skepticism among rural inhabitants previously observed in the UK in 2008 [19]. The process of urbanisation alters natural surfaces and impacts these regulating services, and so urban areas experience increased rainwater surface runoff, increased temperatures and decreased evaporation [66]. As such, urban populations are already facing a range of weather-related risks such as heat waves, air pollution episodes and flooding, and climate change is expected to further compound these problems [9, 67]. Indeed, due to reduced regulating services, the same climate event might result in worse impacts in urban areas when compared to rural areas, at least in some countries; elsewhere, rural wildfires or droughts affecting farming might have much stronger effects on experience [68]. In the UK, urbanites experiencing these climate threats and witnessing first-hand how the hard engineered infrastructure that they rely on in day-to-day life is overwhelmed will likely increase the perceived threat of climate change, and building designers and spatial planners have started to respond to these threats through improved city planning (e.g. green roofs to provide benefits for air quality, mitigating excessive heat and enhancing biodiversity) [67]. In some countries, recent evidence suggests that urban inhabitants accept climate hazards as legitimate reasons for rural-to-urban migration, on a par with other economic, political or social motivations–perhaps also reflecting their perception of the threat of climate change [69].

A further explanation could be provided by differing perceived or experienced levels of resilience between rural and urban inhabitants. Rural people in the UK may experience higher resilience to the impacts of climate change than those in urban, even though objectively measured, the opposite may be the case [70], as subjective and objective measures highlight fundamentally different factors [71]. In Ghana, for instance, high impact of climate change on farmers’ livelihoods is paired with low climate vulnerability and high resilience to climate change [72]. Similarly, field research in the Greenlandic Arctic demonstrates indigenous people’s ability to respond flexibly to climate-based changes to the local environment that provides their livelihood, contrary to claims that they are prime examples of helpless, exposed cultures in crisis [34]. Therefore, one can imagine how rural residents might be more opposed to housing development on a green field site than a change in land use of this greenspace from grassland to marshland (e.g. as might result after increased water levels due to climate change). By contrast, a proposition to transform greenspace (a relatively rare and precious urban resource) to marshland might be met with severe opposition. Given the more direct connections between rural people and the local environment [10], it is likely that rural residents are more used to thinking about nature as part of their daily/weekly routines. Being more disconnected from nature, urban inhabitants may be unused to considering nature in this way and so, when forced to do so by climate change, it is considered more frightening as it is less familiar.

Future work is required to explore this further towards a better understanding of the drivers of this pattern, but also its generalisability to inhabitants of other countries. For example, the adjusted R-sq of our models indicates a large amount of unexplained variance in our data. Thus, indicating that a number of factors other than rurality also contribute to determining the perception of climate change as a threat–future investigations should explore these. Our study was limited by sample size (albeit nationally representative) and restriction to the UK, a country that has so far been spared extreme threats to either urban or rural livelihoods, at least when compared to countries like Pakistan where mitigation strategies are common among farmers [73]. Also, the range of questions included in the survey was limited to allow for rapid data collection, opening up pathways for future studies to address questions around 1) correlations with further demographic, attitudinal and experiential factors, further clarifying the role rurality plays among other factors affecting perception of climate threat; 2) identification of climate skepticism among participants and its effects on responses [19]; and 3) differentiation of diverse facets of place attachment [24], measured by appropriate combinations of free-text and Likert-scale questions. Given the identified concerns around pre-defined scales used to assess place attachment facets, further exploration and validation of diversified approaches to measuring place attachment is highly desirable.

For current purposes, asking one single question has worked well in eliciting rich free-text responses. Our semi-automatic search term based analysis has allowed us to capture and systematically weigh different aspects relevant to place attachment. This procedure resulted in a single measure operationalising strength of place attachment while avoiding the well-known risks of inter-coder discrepancies in traditional content analysis [74], given that the search term lists and weightings are fully transparent and negotiable as such. This paper offers a starting point for such negotiation, suggesting that future research may reveal further nuances and adjustments of the suggested formula. It must also be noted that the automatic part of the procedure carries a different risk, such as of missing context-dependent alternative interpretations of a search term, relevant concepts expressed only indirectly, or failure to detect misspelled items. Although we carefully checked the search results in an iterative process, such errors cannot be entirely eliminated.

## Conclusion

The results of our UK-wide survey showed that although rural people express higher levels of place attachment than urbanites, climate change is viewed as less of a threat by those living in very rural areas than more urban ones. This may have policy implications, especially considering how land use changes and rural farming methods can be decisive in the context of climate change impacts and mitigations [75]. Governments may wish to focus engagement specifically on the most rural areas where climate change threats may not quite have reached people’s awareness, despite enhanced place attachment.

We have offered several possible explanations for this unexpected pattern, including lower levels of awareness of the anthropogenic causes of climate change, lessened first-hand experiences of climate change impacts due to higher levels of regulating ecosystem services, and higher levels of experienced resilience in rural areas. Indeed, these are not mutually exclusive and may be additive (e.g. rural residents may have experienced less severe impacts of climate change, as well as being more resilient to these impacts).

## Supporting information

S1 Table Survey results from our nationally representative sample of 1,071 respondents. (DOCX)

S2 Table The output of the statistical model represented in Eqon 2. This is visually represented in S1 Fig. Note, rurality is an ordinal variable and so the model fits a series of polynomial functions to the levels of the variable: the first is linear (.L), the second is quadratic (.Q), the third is cubic (.C), and the last (^4) is to the power four.
(DOCX)

S3 Table The output of the statistical model represented in Eq 3. Note, rurality is an ordinal variable and so the model fits a series of polynomial functions to the levels of the variable: the first is linear (.L), the second is quadratic (.Q), the third is cubic (.C), and the last (^4) is to the power four.
(DOCX)

S4 Table The output of the statistical model represented in Eq 4. This is visually represented in Fig 1. Note, rurality is an ordinal variable and so the model fits a series of polynomial functions to the levels of the variable: the first is linear (.L), the second is quadratic (.Q), the third is cubic (.C), and the last (^4) is to the power four.
(DOCX)

S5 Table The output of the statistical model represented in Eq 5. This is visually represented in Fig 2 and S2 Fig. Note, rurality is an ordinal variable and so the model fits a series of polynomial functions to the levels of the variable: the first is linear (.L), the second is quadratic (.Q), the third is cubic (.C), and the last (^4) is to the power four.
(DOCX)

S1 Fig An effects plot comparing explanatory variables with the perceived threat of climate change (n = 1,071; Eq 1). Rurality was a significant predictor of perceived threat of climate change and is shown in Fig 1 (S2 Table).
(DOCX)

S2 Fig An effects plot comparing self-defined rurality and wordcount with the place attachment score (n = 1,071; Eq 5). Free text descriptions with higher word count obtained a higher place attachment score (with each additional word raising the score by 0.15; p < 0.001). Rurality was also a significant predictor of place attachment score, with the most rural people having the highest place scores (S3 Table).
(DOCX)
