# Supplementary material for: Place attachment and perception of climate change as a threat in rural and urban areas
Source: PLoS One. 2023 Sep 6;18(9):e0290354. doi: 10.1371/journal.pone.0290354 (PMC10482299; doi:10.1371/journal.pone.0290354)
Supplement: S1 Table — (DOCX) [file pone.0290354.s001.docx]

**S1 Table. Survey results from our nationally representative sample of 1,071 respondents**

| **Rurality** | **Threat** | **Please describe your local area, in terms of what it means to you personally.** | **Gender** | **Age** | **SEG** | **Region** |
| --- | --- | --- | --- | --- | --- | --- |
| 4 | 5 | Not much | Male | 26 | DE | North East |
| 3 | 2 | Close to countryside | Male | 34 | AB | North West |
| 3 | 3 | It’s hone. | Male | 62 | C1 | Scotland |
| 3 | 4 | Local knowledge for help. Readily available countryside for dog walking. Close enough to conurbations if required. | Male | 69 | C1 | East of England |
| 3 | 2 | A town | Female | 21 | C2 | North West |
| 4 | 2 | A garden city surrounded by nature of a forest. | Male | 64 | DE | Scotland |
| 3 | 3 | Border of Town, on high ground, low risk of flooding | Male | 66 | C1 | The South East |
| 3 | 2 | Home | Male | 42 | AB | North East |
| 3 | 4 | Great friendly town | Female | 64 | C2 | The South West |
| 5 | 3 | I love the area I live in, it’s lovely and clean | Female | 58 | AB | North East |
| 3 | 2 | Home | Female | 26 | C2 | East Midlands |
| 5 | 5 | home | Male | 29 | AB | Scotland |
| 5 | 4 | I don't know | Male | 41 | AB | The South East |
| 3 | 3 | Nice | Male | 24 | C1 | The South East |
| 4 | 3 | safe | Female | 31 | DE | East Midlands |
| 5 | 3 | Nothing | Female | 55 | C1 | North East |
| 5 | 5 | A place to live in | Male | 52 | DE | Wales |
| 4 | 4 | Pretty | Female | 26 | C2 | The South East |
| 3 | 1 | Cool | Male | 58 | C2 | North East |
| 3 | 3 | A quiet street. Lots of trees. | Female | 70 | C1 | North West |
| 4 | 3 | Where I live | Male | 25 | DE | North West |
| 1 | 5 | Rural | Female | 74 | C2 | The South West |
| 4 | 3 | Cess pit | Female | 33 | DE | North East |
| 2 | 2 | Too crowded in the summer months | Female | 79 | DE | East of England |
| 4 | 4 | Not sure | Female | 21 | AB | Greater London |
| 3 | 3 | Don’t know | Female | 40 | DE | West Midlands |
| 3 | 3 | Beautiful | Female | 24 | C2 | The South East |
| 3 | 3 | somewhere i live | Male | 54 | C2 | Wales |
| 5 | 1 | Friendly | Female | 62 | C1 | North West |
| 3 | 5 | I live here | Male | 62 | C2 | North East |
| 2 | 3 | Its home | Female | 45 | DE | Wales |
| 1 | 3 | great neighbours | Male | 64 | AB | East Midlands |
| 2 | 2 | Great place to live and close to place of work | Male | 58 | AB | North East |
| 4 | 2 | Lots of Green space to wander in. | Female | 74 | C2 | Greater London |
| 4 | 4 | lovely | Female | 44 | AB | The South East |
| 3 | 2 | It’s lovely, near beautiful countryside | Female | 49 | C2 | North West |
| 3 | 3 | Home | Male | 38 | C1 | North West |
| 5 | 1 | It’s home | Male | 58 | C1 | Scotland |
| 4 | 5 | Unfortunately only an area to live for the time being - will be looking to move soon | Male | 30 | C1 | West Midlands |
| 4 | 3 | A place to live | Female | 46 | DE | North East |
| 4 | 4 | Too many new builds going on and area loosing its green space | Female | 69 | C1 | Greater London |
| 4 | 5 | It is my refuge, with lots of woods and trees to help me feel calm and relaxed | Male | 51 | DE | North West |
| 4 | 3 | Home | Female | 44 | C1 | North East |
| 1 | 2 | Beautiful views, lovely villagers. | Female | 77 | AB | Scotland |
| 3 | 3 | Open countryside on my doorstop and shops are convenient although no proper bus service | Female | 86 | C1 | The South East |
| 2 | 4 | Close enough to civilisation but not too close to cities. I always live where I can hear birdsong:) | Female | 57 | C1 | East of England |
| 4 | 3 | ok at the moment | Male | 61 | DE | The South West |
| 3 | 4 | a place to live | Male | 45 | C1 | Scotland |
| 3 | 3 | I grew up in the area but too much building houses taking over green space now. | Female | 48 | C1 | Scotland |
| 3 | 3 | green space but town like | Male | 47 | C2 | Greater London |
| 2 | 2 | Yes | Male | 24 | C1 | East Midlands |
| 3 | 4 | Live near the sea | Female | 21 | C1 | The South East |
| 2 | 2 | Birth town | Male | 71 | C2 | East Midlands |
| 4 | 3 | Stability | Male | 63 | AB | North East |
| 4 | 4 | I’ve family area | Female | 51 | DE | West Midlands |
| 4 | 5 | HOME | Male | 78 | C2 | The South East |
| 3 | 3 | Quiet, close to the countryside and peaceful | Male | 77 | C1 | East Midlands |
| 5 | 3 | My love my local area | Female | 51 | C1 | The South East |
| 3 | 4 | A place to live, where my house is. | Male | 36 | C1 | East of England |
| 4 | 1 | Home | Male | 47 | AB | North East |
| 4 | 4 | Insecurity over the long term threat to my home. | Male | 66 | DE | East of England |
| 3 | 2 | It doesn't mean a lot to me as I don't see myself here long term | Male | 22 | C1 | The South East |
| 2 | 4 | Beautiful, quiet, traditional | Female | 35 | AB | West Midlands |
| 3 | 3 | Very welcoming and peaceful | Male | 60 | DE | North East |
| 3 | 2 | Rural outlook but close to amenities | Female | 68 | C1 | Greater London |
| 3 | 3 | Best of both worlds. | Female | 46 | C1 | The South East |
| 3 | 2 | Not sure | Female | 23 | DE | The South East |
| 3 | 1 | Small town | Male | 72 | C1 | Scotland |
| 2 | 2 | Home | Female | 70 | DE | North West |
| 3 | 3 | Housing development, on edge of Croydon, but surrounded by met open Land & ASIC. | Male | 75 | C1 | Greater London |
| 2 | 2 | SOMEWHERE TO LIVE | Female | 75 | C2 | West Midlands |
| 1 | 1 | Nothing to say | Male | 61 | C2 | The South West |
| 5 | 3 | Home. First bought house, married & had child from here. Will always be a special place. | Female | 42 | C2 | Scotland |
| 4 | 4 | Somewhere to live | Male | 65 | C2 | North East |
| 5 | 1 | The biggest estate in Norwich so claustrophobic! | Male | 64 | DE | East of England |
| 4 | 3 | Great. | Male | 50 | C2 | North East |
| 4 | 3 | Home | Female | 33 | C1 | The South East |
| 2 | 3 | Lovely and quiet with lovely views. | Female | 60 | C2 | Wales |
| 2 | 3 | Ellesmere is a lovely Georgian town with lovely park and walks. | Male | 57 | DE | Wales |
| 5 | 5 | Home | Female | 24 | C1 | The South East |
| 4 | 3 | Family, friends, history and culture | Male | 35 | C1 | The South East |
| 4 | 2 | on the outskirts of town, near the coast | Female | 63 | C2 | Wales |
| 1 | 3 | Home | Female | 28 | C1 | Greater London |
| 2 | 3 | A lovely place to live | Female | 49 | DE | North East |
| 2 | 2 | Quiet village | Male | 70 | DE | Scotland |
| 3 | 1 | We boo the knee - All Lives Matter | Male | 60 | DE | East Midlands |
| 3 | 3 | A home | Female | 69 | AB | North East |
| 2 | 2 | Its away from London | Male | 78 | C2 | Scotland |
| 5 | 5 | Loud | Female | 40 | AB | Scotland |
| 4 | 2 | Familiar | Female | 55 | AB | Greater London |
| 4 | 1 | Nothing | Male | 24 | DE | The South East |
| 3 | 4 | Sound | Male | 42 | AB | Wales |
| 2 | 1 | Crime | Female | 24 | AB | The South East |
| 2 | 2 | My lovely little rural farming village means the world to me! We’re all related through marriage and we have a fantastic community spirit | Female | 42 | DE | North West |
| 3 | 3 | convenience | Female | 69 | AB | Greater London |
| 2 | 3 | Not much, I wish I still lived where I used to live as in a town I was local to everything I needed. | Female | 37 | AB | East of England |
| 4 | 5 | Beautiful | Female | 35 | C2 | The South East |
| 3 | 2 | Nature | Male | 48 | C1 | Scotland |
| 3 | 2 | Home | Male | 35 | AB | East Midlands |
| 3 | 3 | Home | Female | 34 | DE | East Midlands |
| 4 | 4 | It’s beautiful | Male | 29 | C1 | The South West |
| 3 | 3 | It’s home | Female | 28 | AB | Wales |
| 5 | 1 | very nice area | Male | 77 | C2 | East of England |
| 2 | 3 | A series | Male | 59 | DE | Wales |
| 1 | 2 | Lovely | Female | 42 | DE | The South East |
| 2 | 4 | quiet,peaceful with good wild life | Male | 68 | C2 | East of England |
| 4 | 3 | Air quality reduced because of airport. | Male | 31 | AB | The South East |
| 3 | 4 | Very green and distanced sometimes | Male | 22 | C1 | Greater London |
| 4 | 3 | It is my home, where all my best memories are made | Male | 68 | C1 | North East |
| 5 | 1 | Crap | Male | 47 | AB | Greater London |
| 3 | 3 | ok | Male | 86 | AB | East of England |
| 3 | 3 | Good neighborhood, good range of shops in reasonable distance | Female | 34 | C2 | West Midlands |
| 3 | 3 | Home | Female | 55 | DE | Scotland |
| 4 | 3 | Council has let the town get too run down | Female | 51 | DE | The South East |
| 3 | 2 | Lovely | Female | 53 | AB | East of England |
| 3 | 4 | Quiet friendly estate. | Female | 58 | DE | The South West |
| 4 | 3 | Home | Male | 42 | DE | North West |
| 3 | 2 | Great | Female | 24 | C1 | North West |
| 2 | 3 | Can be quiet and has some incredible scenery within it such as a world heritage site | Female | 28 | C1 | West Midlands |
| 4 | 2 | Home | Male | 23 | AB | North East |
| 1 | 3 | yep | Female | 19 | AB | West Midlands |
| 3 | 4 | Home | Male | 147 | C2 | Scotland |
| 4 | 3 | Nice | Female | 33 | DE | Scotland |
| 3 | 3 | coastal | Male | 65 | AB | Wales |
| 4 | 4 | LARGE TOWN NEAR THE SEA | Male | 51 | DE | Scotland |
| 1 | 2 | Home | Female | 20 | C1 | West Midlands |
| 3 | 4 | Bootiful! | Male | 54 | AB | Wales |
| 1 | 2 | out in wild | Male | 65 | C2 | East Midlands |
| 5 | 3 | I love the local parks | Female | 53 | C1 | Greater London |
| 2 | 3 | Getting rather built up now | Male | 60 | C2 | East of England |
| 4 | 4 | really awful place to live - cant wait to move somewhere else | Female | 22 | AB | North East |
| 2 | 5 | It's quiet and friendly | Male | 58 | AB | East of England |
| 4 | 4 | Personal | Male | 30 | DE | Greater London |
| 3 | 2 | Its OK | Female | 78 | C2 | North East |
| 1 | 1 | Peace, quiet, fresh air. | Female | 53 | AB | Scotland |
| 5 | 5 | Where my family live | Female | 35 | AB | North East |
| 3 | 4 | nice | Female | 23 | DE | Wales |
| 3 | 3 | Friendly | Female | 39 | C1 | The South West |
| 5 | 4 | Convenient | Female | 43 | C2 | The South East |
| 5 | 5 | OK town | Female | 64 | C2 | The South East |
| 3 | 3 | Nice, happy, calm. | Male | 72 | DE | North East |
| 1 | 1 | Lovely | Female | 31 | C2 | The South West |
| 2 | 3 | Villahe | Female | 37 | AB | West Midlands |
| 4 | 1 | Doesnt mean much to me | Female | 24 | C2 | North West |
| 4 | 3 | It's my home town | Male | 32 | C2 | Greater London |
| 3 | 3 | Dual carriageway within 800m one side, countryside the other. | Male | 61 | C1 | East of England |
| 3 | 5 | It’s a lovely quiet village at the moment but a lot of houses are being built on the outskirts and it won’t be the same. I like the quite life. | Female | 73 | DE | The South East |
| 2 | 2 | Nature, love, home, family | Female | 26 | C1 | The South West |
| 3 | 1 | Welcoming | Female | 42 | C2 | North West |
| 4 | 4 | Lots of character, easy to get around | Male | 32 | AB | The South West |
| 5 | 4 | Nice but needs improvements | Male | 29 | C2 | The South West |
| 1 | 3 | No thanks | Female | 48 | C1 | Wales |
| 2 | 3 | Home | Female | 33 | AB | West Midlands |
| 4 | 2 | Perfect balance of mountains, country, city and sea | Female | 41 | C1 | Scotland |
| 5 | 3 | It’s close to my work so easy commute and has a nice garden to relax in. Its also not far from a wide range of amenities. | Male | 46 | AB | Scotland |
| 5 | 3 | Typical low income, built up area | Female | 49 | C2 | Greater London |
| 3 | 4 | Nice area, fields around but the town isn't too far away. | Female | 27 | C1 | North West |
| 3 | 3 | access to countryside from front door but close to the city as well | Female | 41 | AB | North East |
| 1 | 1 | quiet, no shops, no public transport so low risk of covid | Female | 62 | AB | Wales |
| 1 | 3 | Picturesque | Female | 52 | C1 | Scotland |
| 1 | 1 | Home | Male | 51 | C2 | North West |
| 3 | 3 | nice | Male | 84 | C2 | West Midlands |
| 2 | 3 | It means everything to me. I love living here and couldn't imagine living anywhere else. | Female | 75 | AB | The South West |
| 3 | 3 | Community feel | Female | 24 | DE | North West |
| 3 | 3 | Good transport links,best castle in Wales | Male | 65 | AB | Wales |
| 4 | 3 | I love living here, but local council seem to want to turn it into an over populated eyesore of flats! | Female | 47 | C1 | Greater London |
| 3 | 3 | Quiet but close enough to local shops and amenities | Male | 73 | C1 | North East |
| 5 | 3 | I love where I live as there are lots of things to do around the area | Male | 24 | C1 | Scotland |
| 3 | 2 | Countryside | Male | 37 | C2 | West Midlands |
| 5 | 4 | All shops on doorstep but so is the traffic and noise. | Female | 65 | DE | Greater London |
| 5 | 2 | My hometown it meams a lot | Female | 70 | C1 | North East |
| 2 | 3 | Not much | Female | 41 | AB | North East |
| 2 | 3 | Open space green trees everywhere | Female | 53 | C2 | North West |
| 3 | 5 | Sanctuary, moments of peace, calm, nature | Female | 24 | C1 | North West |
| 2 | 3 | Peace | Female | 40 | AB | North East |
| 4 | 5 | Near a public park by River Kennet which floods often throughout the year. | Male | 40 | DE | The South East |
| 4 | 3 | town | Male | 60 | AB | West Midlands |
| 3 | 4 | It is a quiet town, close to beaches and countryside, it suits me totally | Female | 58 | C1 | The South West |
| 3 | 5 | A community that gives me joy | Female | 32 | AB | The South East |
| 3 | 2 | home | Male | 66 | AB | The South East |
| 4 | 2 | Lovely | Female | 21 | AB | The South West |
| 3 | 3 | It's being built up and up and I don't like it. I loved moving here for it's many green spaces which are slowly being taken up with housing. Sad. | Female | 39 | C1 | East of England |
| 3 | 2 | a village | Female | 21 | C2 | West Midlands |
| 5 | 4 | busy | Female | 42 | C2 | Greater London |
| 4 | 3 | my home | Female | 55 | C2 | Greater London |
| 3 | 2 | It's home | Female | 34 | AB | The South East |
| 4 | 3 | Busy | Male | 26 | C1 | Greater London |
| 5 | 5 | Nice, unique area | Female | 29 | C2 | Greater London |
| 5 | 4 | Middle of the city of Cardiff but plenty of parks and green spaces. | Female | 64 | DE | Wales |
| 4 | 4 | Abc | Female | 39 | DE | West Midlands |
| 4 | 1 | Comfortable living. | Female | 74 | DE | North West |
| 3 | 2 | Ok | Male | 49 | C2 | The South West |
| 1 | 1 | Peaceful | Female | 66 | C2 | East of England |
| 5 | 5 | Not really sure | Female | 28 | C1 | East Midlands |
| 2 | 3 | Not sure | Female | 34 | AB | North East |
| 3 | 3 | Beautiful Area | Male | 67 | DE | North West |
| 5 | 5 | M38 8hy | Male | 56 | DE | North West |
| 3 | 2 | Nice | Female | 32 | C2 | East of England |
| 5 | 3 | Shithole | Male | 49 | DE | North East |
| 4 | 5 | An urban area, close to City centre. | Male | 77 | DE | North East |
| 4 | 3 | Community | Female | 49 | C2 | North West |
| 2 | 3 | Everything | Female | 34 | AB | The South West |
| 4 | 5 | Memory's of growing up | Male | 49 | C1 | Greater London |
| 2 | 3 | Nice | Male | 24 | C1 | East Midlands |
| 3 | 3 | home | Male | 71 | C1 | The South East |
| 3 | 4 | Run down, blend of social and private housing, somewhere affordable to live but not where I want to be | Female | 54 | C1 | West Midlands |
| 2 | 3 | Seeing the sea | Female | 61 | AB | North East |
| 1 | 2 | Home | Female | 34 | C2 | North West |
| 5 | 3 | Boring town with no rail link and nothing to do. | Female | 33 | C1 | North West |
| 3 | 3 | It's of great importance to me | Female | 53 | AB | East Midlands |
| 4 | 4 | suburban and nice | Male | 70 | C1 | North East |
| 4 | 3 | clean, but heavily populated. | Male | 80 | C1 | East Midlands |
| 3 | 3 | Lovely | Female | 52 | C2 | East Midlands |
| 3 | 3 | Not a lot. | Male | 34 | DE | Wales |
| 4 | 4 | Home | Female | 22 | AB | West Midlands |
| 1 | 3 | Everything | Male | 45 | C2 | Scotland |
| 3 | 3 | Community driven | Female | 53 | AB | North West |
| 4 | 1 | lots of massage parlours and lovely women | Male | 36 | DE | The South West |
| 4 | 3 | We are close to good shops and to the motorway for work | Female | 33 | AB | East Midlands |
| 5 | 5 | Boss | Female | 68 | DE | North West |
| 2 | 3 | Home | Female | 60 | C1 | East of England |
| 3 | 3 | Lovely, lots of woodland while being not to far from the city | Female | 40 | AB | North East |
| 3 | 3 | happy place | Female | 45 | C2 | West Midlands |
| 3 | 5 | Its my final home. | Male | 52 | AB | East of England |
| 3 | 3 | Decent | Male | 24 | C1 | The South West |
| 5 | 3 | Nothing really | Female | 23 | C1 | The South East |
| 3 | 3 | Peaceful | Male | 40 | DE | Wales |
| 4 | 1 | It's just a place to live! | Female | 60 | AB | North East |
| 2 | 4 | Quite country living | Male | 73 | AB | East of England |
| 3 | 1 | quintessential | Female | 19 | AB | East Midlands |
| 3 | 2 | Semi rural | Female | 35 | C1 | North West |
| 4 | 5 | Like it. | Male | 49 | DE | The South East |
| 4 | 3 | Leafy | Male | 41 | AB | Greater London |
| 5 | 4 | Nice | Female | 41 | C2 | Greater London |
| 3 | 5 | Home | Female | 37 | DE | The South East |
| 4 | 3 | Home | Female | 57 | DE | Greater London |
| 3 | 2 | tidy | Male | 74 | C1 | The South East |
| 4 | 1 | immediate area pleasant, friendly, good neighbours. Wider area run down and ugly, covered in litter and unpleasant | Female | 79 | AB | North West |
| 3 | 3 | small town | Male | 52 | C2 | Scotland |
| 4 | 4 | Sociable | Female | 67 | C1 | The South West |
| 5 | 4 | Busy, crowded want to move. | Male | 64 | DE | The South East |
| 2 | 4 | Family and friends | Male | 23 | AB | The South East |
| 5 | 4 | Within half a mile to the country | Male | 82 | C2 | West Midlands |
| 3 | 3 | good | Female | 38 | C1 | Wales |
| 3 | 3 | Quiet, on edge of small country town. | Female | 72 | C2 | Scotland |
| 2 | 3 | Nice area, but would like to live very rural | Female | 55 | DE | The South East |
| 2 | 4 | The place where I was born, grew up and went to school, first friend were here | Male | 62 | DE | North East |
| 3 | 3 | The community | Male | 53 | AB | Greater London |
| 4 | 3 | Home | Male | 32 | C1 | North East |
| 4 | 4 | Quiet | Male | 72 | C2 | West Midlands |
| 1 | 3 | Quiet. | Male | 49 | C1 | Scotland |
| 5 | 4 | Home | Male | 48 | C1 | Greater London |
| 4 | 5 | It's my own little oasis | Female | 24 | DE | The South East |
| 4 | 4 | It's a peaceful, almost semirurual area which I find very pleasant. | Male | 75 | C2 | The South East |
| 3 | 3 | Food | Female | 31 | AB | Wales |
| 3 | 3 | Nice country town | Male | 45 | AB | East of England |
| 5 | 3 | What I can afford | Female | 54 | DE | North West |
| 4 | 5 | Good | Female | 32 | AB | Greater London |
| 3 | 3 | QUITE VERY FRIENDLY | Male | 52 | C1 | North East |
| 5 | 4 | Uglyville | Female | 59 | C1 | The South East |
| 5 | 3 | Home | Male | 32 | AB | The South West |
| 3 | 3 | Mixture of urban and rural | Male | 55 | DE | West Midlands |
| 4 | 2 | Suburban, quiet, peaceful, makes me feel comfortable and safe | Male | 22 | C1 | North West |
| 4 | 2 | My home | Female | 31 | C2 | North West |
| 2 | 4 | Idyllic | Female | 65 | AB | The South East |
| 2 | 3 | It's green and I like it that way. | Male | 48 | AB | North West |
| 3 | 2 | Good community | Female | 55 | C1 | North West |
| 2 | 2 | Warringto. | Male | 36 | C1 | North West |
| 3 | 3 | great place to live | Male | 67 | C1 | West Midlands |
| 2 | 3 | dont know | Male | 77 | C1 | West Midlands |
| 5 | 5 | rubbish | Male | 75 | C2 | Greater London |
| 3 | 2 | Stupid question | Male | 61 | AB | The South East |
| 4 | 4 | A place surrounded by water that could be flooded | Female | 54 | C1 | The South West |
| 4 | 3 | Overcrowded | Male | 49 | C1 | West Midlands |
| 4 | 3 | Seaside town | Female | 44 | C2 | The South East |
| 3 | 3 | Friendly, pleasant area | Female | 56 | DE | North East |
| 5 | 3 | Just a place to live | Male | 45 | DE | West Midlands |
| 3 | 1 | quiet,backs onto woods | Female | 63 | AB | North East |
| 1 | 3 | Home | Female | 57 | C2 | East Midlands |
| 4 | 3 | It's great | Female | 36 | AB | North East |
| 5 | 3 | Somewhere I live | Male | 45 | AB | Wales |
| 4 | 5 | Love it | Male | 37 | AB | West Midlands |
| 4 | 3 | Close to the countryside | Female | 24 | AB | The South West |
| 5 | 2 | Not very nice | Male | 54 | DE | North West |
| 3 | 2 | its home | Male | 68 | DE | North East |
| 5 | 5 | An area populated by students and young families. | Female | 24 | C1 | North East |
| 4 | 5 | It’s home | Female | 42 | C1 | The South East |
| 4 | 4 | Ok | Male | 66 | C1 | West Midlands |
| 5 | 4 | Wonderful, friendly village. | Female | 70 | C1 | West Midlands |
| 5 | 3 | It's a dump | Male | 53 | DE | North West |
| 3 | 4 | Community | Female | 50 | C1 | North West |
| 2 | 3 | It’s a lovely area to live in because it’s so rural. | Female | 67 | C2 | North West |
| 5 | 2 | Quiet freedom fresh air | Male | 74 | C2 | Scotland |
| 3 | 5 | Peaceful | Female | 34 | DE | The South West |
| 5 | 1 | Flat near friends shops & buses | Female | 64 | DE | The South East |
| 3 | 4 | Awesome | Female | 18 | AB | Greater London |
| 2 | 4 | Lovely place to live | Male | 41 | AB | Wales |
| 4 | 2 | convenient | Male | 66 | AB | East of England |
| 2 | 3 | Village | Female | 67 | AB | East Midlands |
| 5 | 3 | London | Male | 23 | DE | Greater London |
| 2 | 5 | Peace | Male | 32 | DE | The South West |
| 5 | 1 | Couldn't care less | Male | 37 | C1 | The South West |
| 2 | 5 | Moved recently. Doesn't mean much yet | Female | 63 | AB | East Midlands |
| 3 | 2 | Quiet | Male | 73 | C2 | North West |
| 3 | 4 | Home | Male | 60 | C2 | The South East |
| 2 | 3 | It’s my home | Female | 36 | C1 | The South East |
| 3 | 2 | Small town | Female | 29 | DE | East of England |
| 5 | 1 | Safe place to live | Male | 47 | DE | Scotland |
| 5 | 4 | Don’t like it really | Female | 21 | C1 | The South East |
| 4 | 3 | great environment | Male | 76 | AB | East of England |
| 3 | 2 | Its where I grew up | Female | 19 | DE | Scotland |
| 3 | 2 | Home | Male | 31 | C1 | East of England |
| 4 | 3 | Family, friends and community | Female | 24 | C1 | West Midlands |
| 3 | 1 | quite and nice friendly place | Female | 52 | AB | Scotland |
| 1 | 5 | Unspoilt | Female | 77 | C2 | East of England |
| 3 | 2 | makes me happy | Male | 71 | DE | East of England |
| 4 | 3 | Home | Male | 57 | DE | North West |
| 1 | 1 | Picturesque | Male | 55 | DE | North East |
| 4 | 5 | Rubbish | Female | 34 | C2 | Greater London |
| 4 | 4 | Home | Male | 44 | C1 | West Midlands |
| 2 | 2 | Its home | Female | 60 | AB | The South East |
| 5 | 3 | Seaside town with great views and clean air. | Female | 73 | C1 | The South East |
| 1 | 5 | Home | Female | 67 | AB | East of England |
| 3 | 3 | scenic | Female | 65 | C1 | Greater London |
| 3 | 3 | Nearby local shops. Easy to get to work. | Female | 24 | DE | Greater London |
| 3 | 4 | Everything | Male | 61 | AB | North East |
| 3 | 4 | Home | Male | 33 | AB | North West |
| 3 | 2 | Nice eh | Male | 31 | C1 | North West |
| 4 | 3 | Relaxing | Male | 42 | C1 | Greater London |
| 3 | 2 | Love my local area. Everyone looks out for Everyone else | Female | 57 | C2 | West Midlands |
| 3 | 5 | I live next to the sea and the woods and I genuinely can’t imagine living without them both | Female | 29 | C2 | East of England |
| 3 | 3 | nice | Male | 24 | C2 | Wales |
| 4 | 3 | Love it | Female | 46 | C2 | Scotland |
| 2 | 5 | A lot | Female | 40 | C1 | North East |
| 2 | 4 | Large village | Female | 53 | C1 | East of England |
| 3 | 3 | Good | Male | 42 | C2 | North East |
| 3 | 4 | I love where I live. Coast and countryside within minutes from my house, and Dartmoor a short drive away too. Devon is a beautiful county. | Female | 37 | C1 | The South West |
| 4 | 3 | Beautiful coastal region one side, countryside the other | Female | 951 | C1 | The South West |
| 3 | 4 | pretty friendly seaside | Female | 22 | DE | The South West |
| 3 | 2 | Holiday makers | Female | 24 | DE | The South West |
| 3 | 3 | Family | Female | 32 | C2 | The South East |
| 4 | 5 | Quiet, historic, accessible countryside, | Male | 60 | C2 | West Midlands |
| 3 | 3 | live outside of town but not in rural area | Female | 74 | C1 | Greater London |
| 3 | 3 | Great place to be. | Female | 67 | DE | East of England |
| 4 | 5 | No thanks | Female | 34 | AB | The South East |
| 1 | 2 | Moving due to neighbours from hell | Male | 66 | C1 | Scotland |
| 4 | 3 | Doesn’t mean anything to me would leave tomorrow | Female | 66 | C2 | West Midlands |
| 3 | 3 | Central, home, enough open space to get out in fresh air. | Male | 65 | C2 | East Midlands |
| 4 | 3 | Close enough to London, but not too close | Female | 53 | C1 | Greater London |
| 4 | 3 | Friendly residential area. Not too busy | Male | 77 | C1 | North East |
| 2 | 3 | A village in the W. Yorkshire Pennines surrounded by moors and valleys where there are many paths and streams to explore during long walks. | Male | 71 | C2 | North West |
| 4 | 3 | ive only just moved here so not a lot yet | Female | 19 | DE | The South East |
| 3 | 2 | It's a lovely neighbourhood, just outside of a small town centre. Friendly neighbours. | Male | 51 | DE | North West |
| 3 | 3 | Home | Male | 68 | C1 | North West |
| 1 | 3 | Quiet | Male | 62 | DE | The South West |
| 5 | 1 | Becoming more violent | Female | 38 | C2 | West Midlands |
| 2 | 3 | Great but socially isolated | Female | 55 | DE | East of England |
| 4 | 4 | Somewhere I have to live | Female | 55 | DE | North West |
| 4 | 3 | Home | Male | 38 | C1 | The South East |
| 3 | 2 | Peacefull | Male | 45 | DE | West Midlands |
| 5 | 3 | Homely | Female | 30 | AB | Greater London |
| 2 | 3 | Quiet, lot's of wild life, friendly neighbours | Male | 63 | AB | East of England |
| 5 | 4 | Friendly | Male | 49 | DE | North West |
| 4 | 5 | On coast , possibility of severe flooding | Female | 77 | C2 | Scotland |
| 3 | 3 | home | Male | 77 | AB | The South East |
| 5 | 1 | I feel comfortable I'm my area, my family have lived in same part of my town for over 100 years. | Male | 51 | DE | North West |
| 3 | 3 | Easy access to town centre, lots of variety of shops and restaurants. Good public transport options, bus, train, taxi | Female | 24 | C2 | The South East |
| 3 | 3 | home | Male | 83 | AB | North West |
| 5 | 3 | I live here | Male | 31 | DE | North East |
| 3 | 4 | Small quiet area with small local businesses | Female | 36 | DE | Greater London |
| 5 | 4 | Nothing | Male | 41 | C1 | North West |
| 4 | 1 | I live on the coast and enjoy a walk and the scenery every day | Male | 72 | AB | North East |
| 4 | 3 | Urban with good amount of green space. Good amenities | Male | 47 | C1 | Greater London |
| 4 | 3 | Small town | Male | 61 | DE | North East |
| 3 | 2 | It's a calm balance between rural and urban as we live 5 miles from a city and 5 miles from a town a good balance | Male | 59 | DE | West Midlands |
| 3 | 4 | Friendly | Female | 49 | C1 | North West |
| 1 | 3 | Quiet & peaceful | Male | 39 | AB | East of England |
| 4 | 4 | it's alright | Male | 21 | AB | Greater London |
| 2 | 3 | Friendly and great amenities. | Female | 52 | AB | North West |
| 3 | 2 | Scenic | Male | 27 | C2 | The South West |
| 1 | 1 | nice | Male | 54 | AB | East of England |
| 2 | 4 | Relaxing green | Female | 68 | AB | Wales |
| 3 | 3 | It's home | Female | 46 | DE | North East |
| 1 | 5 | Its my piece of beauty | Male | 54 | C2 | East of England |
| 1 | 5 | Home | Female | 24 | AB | The South East |
| 3 | 4 | beautiful coastal town live at sea level so higher tides would wipe out the whole coastal area | Male | 60 | DE | East of England |
| 3 | 4 | Love mix of countryside and town | Female | 33 | AB | North West |
| 2 | 3 | Unspoilt | Female | 41 | AB | Wales |
| 3 | 2 | semi rural with transport links that could be improved. | Male | 64 | DE | North East |
| 3 | 4 | A small village | Female | 24 | AB | The South West |
| 2 | 4 | A community. | Female | 66 | DE | Scotland |
| 4 | 3 | clean and friendly, | Male | 84 | DE | West Midlands |
| 2 | 2 | I would like to see the area have a bright future for my daughter | Female | 35 | AB | Scotland |
| 2 | 4 | I live in a rural village in Norfolk and value the peace and quiet as well as the surrounding countryside which is good for my mental and physical health as I enjoy cycling in quiet country lanes. I am naturally opposed to any developments which may harm the countryside and wildlife. We have had several hundred new houses built in our village in recent years and more are planned as are thousands more in nearby towns. Once we lose our countryside it is lost forever. | Male | 74 | C1 | East of England |
| 2 | 3 | Small friendly | Male | 67 | C2 | East of England |
| 3 | 3 | Quiet and friendly | Female | 23 | C2 | North East |
| 2 | 1 | It is a small village with no shops or post office, 3public houses a gin distillery and a tea garden. There are large meadows on both sides of the river that divides this village from the edge of Cambridge. This area is a highly sort after area to live in. | Male | 77 | C2 | East of England |
| 3 | 3 | Home | Male | 42 | C1 | North East |
| 3 | 3 | Village | Female | 48 | DE | West Midlands |
| 3 | 3 | Lots | Male | 39 | C1 | North East |
| 5 | 1 | Practical with all the amenities needed. Moderately safe. | Female | 69 | DE | The South East |
| 5 | 3 | Urban but close to park | Male | 66 | C2 | The South West |
| 2 | 4 | Where I live, under the same town/village name as my address | Male | 32 | AB | West Midlands |
| 4 | 4 | All my family are here an there's lots of opportunities or young people | Female | 23 | AB | Wales |
| 3 | 2 | Housing estate but with fields very clos by, lots of safe walking areas. | Female | 30 | DE | The South West |
| 2 | 3 | Open countryside with woods | Female | 67 | DE | Scotland |
| 3 | 3 | Where I grew up | Female | 30 | C2 | East Midlands |
| 3 | 3 | Lovelly | Female | 36 | C1 | Scotland |
| 3 | 2 | A dump | Male | 34 | AB | Greater London |
| 2 | 3 | Always be home where everyone knows everyone | Female | 24 | C2 | Wales |
| 4 | 3 | Home | Female | 32 | DE | The South East |
| 3 | 3 | Beautiful | Female | 49 | AB | North West |
| 4 | 4 | Very nice area to live in | Male | 71 | DE | The South West |
| 5 | 4 | It's the place that I live | Female | 49 | C1 | Scotland |
| 3 | 1 | A wonderful place to live… | Female | 65 | AB | East of England |
| 5 | 3 | Built up with some green space | Female | 46 | C1 | The South East |
| 3 | 4 | Home | Female | 49 | C1 | North East |
| 3 | 3 | It's home | Female | 32 | DE | Wales |
| 2 | 1 | Not sure | Female | 41 | DE | West Midlands |
| 4 | 1 | Lived in the area for 20+ years, but it's gone downhill rapidly in the last 10 and now I am eager to leave for a more rural location | Female | 24 | C2 | The South East |
| 2 | 3 | Nothing | Male | 67 | DE | Scotland |
| 5 | 3 | Comfort | Female | 26 | C1 | The South East |
| 4 | 3 | Busy | Female | 42 | C1 | The South East |
| 5 | 3 | Flooding | Female | 44 | AB | The South East |
| 5 | 3 | a lovely welcoming part of town with the feel of a village | Female | 54 | C1 | East Midlands |
| 1 | 1 | Small | Male | 33 | DE | The South West |
| 3 | 4 | Home | Male | 46 | C2 | The South East |
| 3 | 4 | Semi rural, nice surroundings, | Male | 66 | C2 | The South East |
| 3 | 5 | Increasing air pollution, reduction in green space, rising water levels, more erratic weather | Male | 33 | C1 | North West |
| 5 | 5 | ?Shithole? | Female | 20 | DE | North West |
| 3 | 2 | I like living in Chelmsford but I have only been here 3 years so I have no attachment | Female | 23 | C1 | The South East |
| 2 | 2 | A refuge of calm, with natural beauty, interesting heritage and architecture and services provided by passionate local people. | Male | 44 | C1 | The South West |
| 1 | 3 | I wouldn't live anywhere else. Peaceful and uninvaded. | Female | 98 | AB | East of England |
| 1 | 2 | It's beautiful farmland on a landed gentry estate safe calm and friendly. | Female | 74 | C1 | East Midlands |
| 5 | 5 | Home | Male | 35 | DE | Greater London |
| 4 | 4 | no | Male | 50 | AB | The South East |
| 3 | 5 | Peaceful | Female | 45 | DE | West Midlands |
| 4 | 3 | It is a nice area | Female | 34 | DE | North West |
| 5 | 3 | Shit | Female | 35 | C2 | Wales |
| 2 | 5 | Home | Male | 63 | C1 | The South West |
| 5 | 5 | Great community and amazing green space | Female | 24 | AB | Scotland |
| 3 | 2 | It's easy access to the coast, countryside and roads to get elsewhere | Male | 63 | AB | East of England |
| 3 | 3 | Home. | Male | 67 | AB | North East |
| 3 | 3 | Countryside, wildlife, peace and quiet | Female | 35 | C1 | The South East |
| 3 | 5 | Home | Male | 60 | C1 | East of England |
| 5 | 3 | Just a place to live | Male | 74 | C1 | North East |
| 2 | 5 | lovely, peaceful(ish), prone to flooding | Female | 62 | C1 | West Midlands |
| 4 | 5 | homely trees | Male | 63 | C1 | East of England |
| 3 | 2 | home | Female | 59 | AB | East of England |
| 2 | 4 | It's where I live | Male | 55 | DE | Greater London |
| 4 | 2 | Home is where your heart is. | Male | 77 | C1 | Wales |
| 5 | 4 | New | Female | 23 | C1 | Greater London |
| 3 | 3 | Got f | Female | 45 | DE | East Midlands |
| 3 | 5 | Quiet, peaceful and very flat | Male | 40 | C1 | East of England |
| 2 | 4 | county where i've grown up | Male | 38 | C2 | East Midlands |
| 3 | 5 | Small town with countryside on our doorstep | Male | 64 | C2 | North East |
| 2 | 4 | Ball bags | Male | 40 | DE | West Midlands |
| 2 | 2 | Beautiful, historic | Male | 24 | AB | West Midlands |
| 2 | 3 | Semi rural relaxed town situated in Gateshead. About 5 minutes from the river Tyne. Beautiful nature and fresh air. | Female | 24 | DE | North East |
| 4 | 2 | A nice area near the city but far enough away to have our own green space for nice quiet waks | Female | 26 | DE | North West |
| 3 | 1 | Everything | Female | 22 | AB | The South East |
| 3 | 3 | village | Male | 36 | C2 | East Midlands |
| 5 | 2 | Run down | Male | 33 | AB | Greater London |
| 3 | 3 | Village near the sea. I've been brought up here, this is home. | Female | 28 | C2 | Wales |
| 5 | 4 | City | Female | 32 | C1 | East Midlands |
| 2 | 3 | Secluded but no amenities at all. | Male | 54 | DE | East of England |
| 4 | 3 | Best of urban and seaside | Male | 59 | AB | Scotland |
| 2 | 1 | no | Male | 53 | DE | East Midlands |
| 3 | 3 | Small village, twenty five minutes from town and the beach. Everyone knows most people. Very friendly p,ace to live | Female | 78 | C2 | Scotland |
| 5 | 1 | Means nothing just a dump i would like to leave | Male | 63 | DE | East Midlands |
| 5 | 4 | Shithole | Female | 63 | C2 | West Midlands |
| 2 | 3 | Peace and quiet with an active friendly community spirit | Male | 68 | C2 | The South West |
| 4 | 5 | safe and fun place to live | Female | 21 | AB | West Midlands |
| 3 | 3 | My local area is currently a joke due to mayor of London’s road narrowing causing constant traffic making it impossible for disabled persons to get around | Female | 44 | AB | Greater London |
| 4 | 2 | Pleasant, not too urban, but within easy reach of most needs. | Male | 74 | C1 | North West |
| 4 | 3 | woods and parks near by | Male | 65 | C2 | West Midlands |
| 5 | 5 | Its my home | Male | 53 | AB | West Midlands |
| 3 | 2 | Home | Male | 38 | C2 | Greater London |
| 3 | 5 | Home | Female | 55 | C1 | East of England |
| 4 | 3 | nice and safe | Female | 23 | C2 | Greater London |
| 4 | 3 | Very nice area | Male | 38 | C1 | North West |
| 3 | 2 | Beautiful beach walks | Female | 70 | AB | East of England |
| 3 | 2 | A community. | Female | 50 | C1 | East of England |
| 3 | 3 | It’s alright | Female | 22 | C2 | The South West |
| 2 | 1 | Simple living | Male | 33 | AB | Scotland |
| 3 | 1 | Home | Female | 32 | C1 | North East |
| 4 | 4 | Flooding | Female | 61 | C1 | Scotland |
| 5 | 3 | Busy | Female | 27 | DE | North East |
| 5 | 5 | It’s greenery is disappearing along with the wild life | Male | 24 | AB | Scotland |
| 3 | 3 | No | Female | 46 | C2 | East Midlands |
| 2 | 3 | peaceful | Male | 54 | C2 | The South West |
| 5 | 2 | Inner city london! | Female | 46 | C1 | The South West |
| 3 | 3 | good facilities, good transport links, area of SSI on the doorstep | Male | 74 | AB | Greater London |
| 2 | 3 | Steelworks town | Female | 24 | C1 | North East |
| 2 | 5 | The place I've grown up, moved away from and then come back to to start my own family. | Male | 24 | AB | The South East |
| 3 | 3 | Lovely | Female | 39 | C2 | North West |
| 5 | 3 | Good | Male | 44 | DE | North East |
| 3 | 4 | It’s my home | Female | 25 | AB | The South West |
| 5 | 2 | Suburban London | Male | 36 | AB | Greater London |
| 4 | 3 | it's a community | Female | 46 | C1 | West Midlands |
| 3 | 3 | Safe | Female | 33 | AB | The South East |
| 5 | 5 | Hopefully going places with regeneration | Female | 65 | C1 | Greater London |
| 4 | 4 | Coastal idle | Male | 69 | AB | North East |
| 4 | 4 | Quite and safe | Male | 32 | DE | Scotland |
| 4 | 3 | Outskirts of town not far from countryside | Female | 74 | AB | Greater London |
| 3 | 3 | It's okay | Male | 31 | C2 | East Midlands |
| 4 | 3 | Happy to live in this area. | Male | 69 | DE | Greater London |
| 3 | 3 | Beautiful | Male | 61 | C2 | The South East |
| 5 | 2 | Outer London, with parks but more and more properties are being built making the roads, hospitals, doctors schools and utilities unable to cope. No consideration of the needs for infrastructure requirements is given when new builds are made. | Male | 82 | AB | Greater London |
| 4 | 3 | Home | Female | 29 | C1 | East Midlands |
| 1 | 4 | rural | Female | 62 | C1 | West Midlands |
| 4 | 3 | Well, I live here. | Female | 24 | DE | Greater London |
| 3 | 3 | Not sure | Female | 58 | C1 | North East |
| 2 | 3 | I was born in a small town and now it's turned into a bigger town in just 5 years with way too many new builds and infrastructure. It's a pretty place tho' | Female | 54 | C2 | The South East |
| 3 | 2 | Landscape and coastal | Female | 53 | C1 | North East |
| 5 | 5 | Small city | Female | 42 | AB | North West |
| 4 | 3 | lowkey rich white neighbourhood, little bit of crime, roads are kind of rubbish but lots of green space around here, as in parks | Female | 19 | DE | Greater London |
| 3 | 3 | Nice and quiet | Male | 60 | DE | North East |
| 3 | 2 | Really like living there. | Male | 48 | AB | Greater London |
| 3 | 4 | No | Female | 22 | DE | North West |
| 4 | 5 | Neglected | Male | 47 | DE | East Midlands |
| 3 | 1 | Happy | Male | 65 | DE | North West |
| 4 | 5 | Bad | Male | 30 | C1 | West Midlands |
| 4 | 3 | Lovely green area and great community | Male | 40 | C2 | East Midlands |
| 3 | 2 | Somewhere to live | Male | 73 | AB | North East |
| 3 | 2 | Nice green spaces | Female | 57 | C1 | Greater London |
| 5 | 3 | I love my house and garden but HATE my area. It is rife with yobs, fly tipping, general rubbish dumping and nowhere nice to take a run or walk. Please crane my house to the countryside!!! | Female | 72 | C1 | East Midlands |
| 5 | 3 | Convenient | Male | 55 | AB | Wales |
| 3 | 2 | Not a lot | Female | 21 | DE | The South West |
| 3 | 5 | we need to protect what green spaces we have left | Male | 39 | C1 | Scotland |
| 5 | 3 | City - love it | Female | 21 | DE | North East |
| 3 | 3 | It's nice | Male | 34 | AB | North East |
| 5 | 3 | Easy access, fun, things to do | Female | 43 | C2 | North West |
| 3 | 2 | Residential | Female | 24 | C1 | Greater London |
| 3 | 4 | it’s fine | Female | 26 | AB | East of England |
| 1 | 2 | Full of family and a safe place | Male | 24 | C2 | The South East |
| 3 | 5 | Home | Female | 53 | C2 | East of England |
| 4 | 1 | Love it | Male | 42 | AB | North East |
| 2 | 4 | Home | Male | 45 | AB | The South East |
| 2 | 4 | Yywy | Female | 20 | DE | The South East |
| 1 | 5 | Home | Male | 68 | AB | West Midlands |
| 4 | 2 | Home | Male | 31 | AB | Greater London |
| 3 | 3 | It’s a quiet and close knit community where most people know each other | Female | 21 | DE | North West |
| 5 | 3 | Home | Female | 25 | AB | Wales |
| 3 | 5 | Over population | Female | 47 | AB | Greater London |
| 4 | 3 | I've leaved here all my life. | Male | 63 | DE | West Midlands |
| 4 | 5 | Creativity, connection to the sea and nature | Male | 22 | DE | The South East |
| 4 | 4 | Busy, bustling. Family close by | Female | 34 | C2 | The South East |
| 5 | 4 | Where me any my family live | Female | 33 | C1 | Greater London |
| 3 | 4 | Too C/conservative | Male | 46 | C1 | The South East |
| 2 | 3 | countryside | Female | 61 | C1 | The South West |
| 3 | 4 | Love it | Female | 24 | C2 | The South East |
| 4 | 1 | No | Male | 39 | DE | North West |
| 3 | 1 | Noisy | Male | 32 | AB | North East |
| 3 | 4 | Semi-Rural but under threat of airport expansion | Female | 57 | AB | Greater London |
| 3 | 4 | love it | Male | 77 | C2 | West Midlands |
| 3 | 1 | is fine | Male | 51 | AB | The South East |
| 3 | 3 | Cool | Female | 23 | AB | West Midlands |
| 3 | 4 | cool | Female | 24 | DE | The South West |
| 3 | 4 | Home | Male | 23 | C1 | East Midlands |
| 5 | 3 | Urban | Female | 62 | AB | Greater London |
| 4 | 3 | Friendly | Female | 23 | AB | Greater London |
| 3 | 3 | Quiet | Female | 44 | DE | Scotland |
| 4 | 4 | Lovely | Male | 39 | DE | Scotland |
| 4 | 3 | Modest | Male | 41 | C2 | East of England |
| 3 | 3 | Nice seaside town | Male | 67 | C1 | East of England |
| 2 | 3 | good | Female | 24 | DE | The South West |
| 2 | 5 | It’s home | Female | 69 | AB | East of England |
| 3 | 3 | Homely , friendly , mainly peaceful | Male | 61 | C2 | North East |
| 4 | 1 | Great | Female | 46 | DE | North East |
| 3 | 3 | Lovely town with a good sense of community and most of what we need on our doorstep, but surrounded by beautiful countryside. Very happy to bring my children up here and feel lucky. | Female | 40 | C1 | East of England |
| 3 | 2 | Home | Female | 54 | AB | East Midlands |
| 2 | 3 | Great! | Male | 62 | AB | The South East |
| 3 | 4 | Way to much new builds going on and green spaces disappearing and amenities not meeting the growing population. | Female | 44 | C2 | Greater London |
| 2 | 4 | natural space, very touristy, open and rural | Female | 21 | C2 | Wales |
| 4 | 4 | Not much | Male | 45 | AB | Greater London |
| 4 | 3 | Home | Female | 28 | C1 | Greater London |
| 2 | 2 | Sea view one way, country view the other, what more could one want. | Male | 68 | C2 | The South West |
| 5 | 3 | Nothing | Male | 43 | AB | Greater London |
| 3 | 2 | Quiet | Male | 66 | C2 | The South East |
| 3 | 3 | Home | Female | 47 | DE | West Midlands |
| 3 | 5 | global warming is bad for everyone | Female | 22 | DE | The South West |
| 4 | 3 | Don't know | Male | 51 | C2 | East of England |
| 4 | 3 | Neighbourhood | Male | 67 | AB | West Midlands |
| 3 | 4 | Recognisable,Safe, Memories, lots of trees and birds etc | Female | 53 | AB | East of England |
| 2 | 2 | Love it | Female | 32 | C2 | North East |
| 1 | 1 | Everything | Female | 54 | AB | The South East |
| 2 | 2 | nice safe country village | Female | 62 | C2 | The South West |
| 3 | 4 | Country side close to city | Female | 24 | C1 | West Midlands |
| 3 | 5 | Semi rural peaceful | Female | 53 | C2 | Scotland |
| 4 | 5 | Ever changing from semi rural to burgeoning edge of town | Male | 47 | DE | West Midlands |
| 5 | 2 | Hefty | Male | 39 | AB | Greater London |
| 4 | 3 | A dive | Female | 32 | DE | East Midlands |
| 3 | 3 | Home | Male | 45 | C1 | Scotland |
| 3 | 3 | Very friendly, lots of green space within a short walk, buses every 15 minutes, small local shops and larger stores in town. I love it here and am so pleased I moved here 6 years ago. | Female | 70 | C2 | The South East |
| 2 | 5 | It's the centre of my physical universe; it's home... | Female | 56 | C1 | Wales |
| 3 | 3 | Full of nature in my garden! It’s anaIng and changes my whole day | Male | 34 | C2 | North West |
| 1 | 1 | It's an Island not only of my mind but also Actually. | Male | 77 | C1 | East of England |
| 3 | 4 | Home | Male | 36 | AB | North East |
| 2 | 4 | degradation | Male | 40 | C2 | The South West |
| 3 | 3 | Home | Female | 43 | DE | Scotland |
| 3 | 4 | Wonderful place to live, by the sea with woods and countryside close by | Female | 71 | AB | North East |
| 4 | 3 | Relaxing, safe place to live in. | Female | 25 | C1 | Greater London |
| 3 | 2 | Home | Male | 41 | C1 | North East |
| 3 | 2 | Precious | Female | 61 | C1 | West Midlands |
| 4 | 3 | A nice area to live in. | Male | 63 | DE | The South East |
| 3 | 3 | safe home | Male | 67 | AB | North East |
| 1 | 2 | Could be better. | Male | 48 | DE | North East |
| 3 | 3 | Home | Male | 35 | AB | North West |
| 2 | 2 | Home away from the busy life of towns.. | Female | 68 | C1 | The South West |
| 5 | 3 | It could be better, but it’s home. That being said, I would leave the area. | Male | 32 | C1 | The South East |
| 2 | 2 | it's a nice place | Male | 31 | AB | East Midlands |
| 4 | 5 | Climate change is really important | Female | 23 | DE | The South East |
| 5 | 4 | It's where I live. | Male | 49 | C1 | North East |
| 1 | 3 | Being close to nature | Female | 41 | AB | East Midlands |
| 4 | 3 | Safe | Female | 44 | C1 | The South East |
| 3 | 1 | what | Male | 31 | AB | North East |
| 3 | 4 | home | Male | 42 | C2 | The South West |
| 4 | 3 | Quiet but close to amenities | Female | 65 | C2 | East of England |
| 2 | 3 | Right on the doorstep of some beautiful green space | Female | 47 | C2 | North East |
| 2 | 3 | Community and village life | Female | 43 | C1 | East Midlands |
| 4 | 4 | Lovely | Female | 66 | AB | North East |
| 5 | 5 | London | Male | 34 | AB | Greater London |
| 5 | 2 | Very busy with loads of recycling bins | Male | 58 | DE | The South East |
| 3 | 2 | Quiet and picturesque | Male | 64 | C1 | East of England |
| 5 | 5 | Gloucester | Male | 35 | C1 | The South West |
| 4 | 3 | Very easy to obtain all items I require and close to green space, easy for transport. | Male | 81 | C1 | East Midlands |
| 1 | 1 | Country | Male | 41 | C1 | North East |
| 4 | 4 | Lots of houses but don’t have to go far for green spaces | Male | 68 | DE | North West |
| 4 | 1 | Beautiful horticulture | Male | 61 | AB | North West |
| 4 | 5 | I love it | Male | 41 | C2 | North East |
| 4 | 3 | Where I live | Male | 33 | AB | North West |
| 5 | 5 | Destruction of natural wildlife populations and habitat  Overcrowding and pollution | Female | 61 | C2 | West Midlands |
| 4 | 2 | LIKE IT | Male | 33 | C1 | The South East |
| 5 | 3 | Home | Male | 45 | C1 | The South East |
| 3 | 4 | Important | Female | 67 | C1 | The South East |
| 5 | 3 | Nice quiet and green. | Male | 59 | DE | North East |
| 3 | 3 | quiet and mostly English people. | Female | 74 | C2 | The South East |
| 3 | 3 | Lots for my child to do | Female | 44 | C2 | North East |
| 3 | 3 | lovely | Male | 64 | DE | East of England |
| 3 | 3 | No | Female | 36 | C1 | East of England |
| 5 | 5 | Home | Male | 29 | AB | Greater London |
| 5 | 3 | It's a lovely no crime zone and close to the shops | Male | 45 | C1 | West Midlands |
| 3 | 3 | Very pleasant normally but with shielding for most of lockdown, I have not ventured out enough to note any significant changes. | Male | 71 | DE | Greater London |
| 4 | 4 | lots | Male | 24 | AB | North West |
| 3 | 4 | sea levels | Male | 52 | C1 | East of England |
| 2 | 2 | I live here. | Male | 50 | AB | Wales |
| 2 | 3 | Peaceful | Female | 25 | AB | The South East |
| 5 | 5 | Having lived here for 54 years alongside the river Taff with tree lined   streets it is very pleasant and peaceful. | Male | 79 | C1 | Wales |
| 3 | 4 | Good neibours | Male | 73 | DE | East of England |
| 2 | 4 | Sheep, cattle, farmland close by but several major motorways easily accessible when required. Best of both world (so long as they stop building new developments very close by) | Female | 58 | C1 | West Midlands |
| 2 | 5 | Getting to built up | Male | 59 | DE | East of England |
| 5 | 4 | Friendly | Female | 41 | C1 | Scotland |
| 3 | 2 | Local town | Male | 52 | AB | North East |
| 4 | 3 | It's friendly | Female | 42 | C1 | East Midlands |
| 3 | 3 | Peaceful | Female | 22 | C1 | West Midlands |
| 4 | 5 | Okay | Male | 63 | AB | Scotland |
| 3 | 3 | A lot | Male | 33 | AB | Greater London |
| 2 | 3 | Small tourist town.  Nothing much has changed here in 34 yrs i have been here. | Female | 57 | DE | Scotland |
| 1 | 3 | Home | Male | 49 | C1 | Scotland |
| 3 | 3 | Lots of nature and important to protect it | Male | 37 | C1 | Greater London |
| 2 | 1 | trees mountains what more is there to say | Male | 59 | DE | Wales |
| 2 | 3 | Beaches | Male | 51 | C1 | North West |
| 4 | 4 | Interesting | Male | 66 | AB | East of England |
| 2 | 3 | A little oasis not too far from local amenities | Male | 71 | AB | The South East |
| 5 | 4 | a lot of good friends | Male | 56 | DE | The South East |
| 2 | 1 | Beautiful | Male | 60 | DE | The South West |
| 4 | 3 | No thanks | Male | 39 | C1 | North East |
| 3 | 1 | Quiet leafy suburb | Male | 44 | DE | North West |
| 4 | 3 | Great | Male | 42 | C2 | North East |
| 2 | 5 | My home | Male | 75 | AB | North East |
| 3 | 1 | My home. | Male | 78 | DE | The South East |
| 1 | 2 | It is a beautiful place to live with lots wildlife on the door step hhedgehogs, badgers, foxes, otters, stoats, weasels, trout in the nearbye river and lots of birds including buzzards, red kite, sparrowhawks and owls. Bats to im woken by birdsong every morning i wouldn't want to live anywhere else | Male | 47 | DE | Wales |
| 4 | 2 | Ok | Female | 31 | C1 | East of England |
| 4 | 5 | No | Female | 22 | AB | North West |
| 3 | 2 | home | Male | 34 | C1 | Greater London |
| 4 | 3 | Nice | Male | 50 | DE | East of England |
| 2 | 3 | Nice countryside. | Male | 63 | C2 | Scotland |
| 3 | 3 | Precious | Male | 60 | AB | North East |
| 4 | 3 | Lots of parks around but also new builds popping up everywhere mean it’s not as nice as it used to be | Female | 43 | C2 | Greater London |
| 2 | 3 | My village, where I have always lived- somewhere that is familiar to me. | Female | 20 | AB | East of England |
| 4 | 4 | Flooding will become an issue on a more frequent basis. | Male | 30 | C1 | The South East |
| 2 | 1 | Beautiful | Male | 51 | C2 | Wales |
| 3 | 4 | A rural village which is being subjected to over development of new houses | Male | 75 | AB | East of England |
| 5 | 1 | council | Male | 57 | DE | North West |
| 3 | 5 | Causing pollution and destroying the planet | Female | 59 | AB | North East |
| 5 | 3 | City centre | Male | 56 | DE | Scotland |
| 4 | 5 | Home | Male | 22 | AB | Wales |
| 4 | 2 | home | Female | 66 | C1 | The South East |
| 5 | 3 | Wonderful | Female | 67 | DE | Scotland |
| 2 | 4 | Great. | Female | 66 | C1 | The South East |
| 3 | 2 | Home | Female | 34 | C1 | North East |
| 3 | 3 | Home | Female | 47 | C2 | The South East |
| 3 | 5 | Nice | Male | 40 | C1 | East of England |
| 1 | 1 | Peace, quiet and tranquility flat farmland. | Male | 61 | AB | East of England |
| 2 | 3 | Safe, natural | Male | 30 | AB | East of England |
| 5 | 5 | No | Female | 49 | DE | The South West |
| 3 | 4 | Quiet a BBC d peaceful | Male | 70 | C2 | North East |
| 3 | 4 | Home, safe, green | Male | 28 | C1 | Greater London |
| 5 | 5 | Noisy | Male | 131 | C1 | Greater London |
| 4 | 2 | Good neighbourhood | Female | 71 | C1 | Greater London |
| 3 | 4 | Great | Female | 69 | DE | North East |
| 2 | 4 | Nice quiet area | Male | 34 | C1 | The South West |
| 3 | 2 | Love it! | Male | 54 | AB | East of England |
| 4 | 2 | Suburban artsy part of Cardiff, media centric lot's of welsh spoken here as a status symbol. I love it but will sadly have to move to a less desirable area soon. | Male | 43 | AB | Wales |
| 4 | 4 | Town | Female | 29 | DE | West Midlands |
| 4 | 5 | Hone | Male | 33 | DE | East Midlands |
| 1 | 5 | Quiet | Male | 31 | AB | The South West |
| 4 | 3 | A place to live | Male | 61 | C1 | Greater London |
| 5 | 2 | A very enviromentally friendly area. | Male | 81 | AB | Greater London |
| 2 | 5 | Beautiful area and biologically diverse. | Female | 50 | AB | The South West |
| 2 | 4 | Nice quiet village | Male | 45 | C1 | East of England |
| 2 | 5 | nature. | Male | 49 | C2 | West Midlands |
| 3 | 2 | Safe | Female | 67 | DE | East Midlands |
| 5 | 3 | A Dump | Female | 33 | AB | Greater London |
| 4 | 4 | It is my home, my childhood and my memories | Female | 23 | AB | The South East |
| 5 | 2 | Great place to live | Male | 40 | C1 | West Midlands |
| 3 | 3 | Special | Male | 53 | DE | Wales |
| 3 | 3 | Village | Female | 31 | DE | Scotland |
| 3 | 2 | Away from city crap | Male | 34 | C2 | East Midlands |
| 5 | 4 | Good neighbors and friendly local community. | Female | 60 | DE | Scotland |
| 4 | 1 | not a lot | Male | 73 | DE | East Midlands |
| 4 | 3 | It’s safe | Female | 32 | AB | East Midlands |
| 5 | 5 | Everything | Female | 32 | C1 | Scotland |
| 3 | 3 | not a lot | Male | 58 | DE | East Midlands |
| 4 | 2 | Unfortunately not much, I just live and work here. | Male | 30 | AB | Greater London |
| 2 | 2 | No | Male | 28 | DE | Scotland |
| 5 | 1 | Love it | Female | 30 | C1 | North East |
| 4 | 5 | It's where I live. Climate change will be the death of everyone regardless of where we live. | Male | 38 | DE | North West |
| 3 | 4 | Relaxing | Female | 46 | C2 | The South West |
| 3 | 2 | A nice quiet small town | Female | 67 | DE | East Midlands |
| 2 | 3 | It is a rich area of native animals vital for the environment. | Male | 74 | C1 | The South West |
| 1 | 2 | National Park | Male | 54 | AB | North East |
| 3 | 4 | A perfect mix of coastline & ocean | Female | 43 | DE | East of England |
| 4 | 4 | I love the seafront - the smells, what you can hear, and the atmosphere. I will struggle to live anywhere but by the sea | Female | 30 | C1 | The South East |
| 2 | 5 | Sadly it is being built upon and all green fields are not safe | Female | 53 | C1 | The South West |
| 2 | 4 | Beautiful | Female | 66 | C1 | North West |
| 4 | 5 | Just outside of London, it’s buzzing and thriving and safe. Green spaces with excellent transport links | Female | 29 | AB | The South East |
| 3 | 2 | fuck off | Male | 42 | C1 | North West |
| 1 | 1 | Small village five miles from nearest town. Away from the main road it is quiet and peaceful and it is a pleasure to hear the dawn chorus. | Male | 73 | C1 | East Midlands |
| 5 | 3 | Noisy concrete jungle | Male | 47 | C1 | Greater London |
| 3 | 3 | Auchinleck | Male | 46 | C1 | The South East |
| 3 | 3 | A beautiful place by the beach. | Male | 38 | AB | North West |
| 4 | 3 | Beautiful place to live | Male | 23 | AB | The South West |
| 1 | 3 | Quiet life | Female | 36 | DE | East Midlands |
| 3 | 3 | A village that has lots of farm land surrounding, but not too far from the local town | Female | 42 | AB | Greater London |
| 5 | 4 | Not great | Female | 29 | C2 | Greater London |
| 2 | 3 | Cosy, calm and a sanctuary | Male | 34 | AB | The South East |
| 5 | 1 | nice place to live | Male | 64 | C2 | The South West |
| 3 | 2 | Village community with increased housing development | Female | 66 | AB | North East |
| 3 | 4 | It's home and I don't want it to change | Female | 24 | C1 | The South East |
| 5 | 3 | Home | Male | 52 | AB | Greater London |
| 3 | 1 | good balance of city, sea and mountains | Female | 24 | DE | Wales |
| 4 | 5 | Nothing. Town of junkies. | Female | 31 | DE | North West |
| 5 | 3 | where I live | Male | 39 | C1 | Greater London |
| 4 | 3 | Plenty of Green Countryside and the animals about | Female | 45 | C2 | Scotland |
| 3 | 3 | Small offshoot of local town with good amount of trees and coastline | Female | 66 | C1 | The South East |
| 4 | 4 | No | Female | 31 | C1 | North East |
| 3 | 3 | I like the country park and I am passionate it is there for all | Female | 53 | AB | East Midlands |
| 4 | 2 | Semi-suburban. Area of choice | Male | 52 | AB | The South East |
| 3 | 5 | dead when it rains | Male | 76 | DE | The South East |
| 4 | 4 | Great balance between urban and green spaces | Female | 41 | DE | North West |
| 3 | 2 | Precious | Male | 46 | DE | Wales |
| 3 | 3 | Grew up here | Female | 41 | DE | North West |
| 2 | 1 | Calm | Female | 31 | DE | Scotland |
| 4 | 1 | Almost ideal. | Male | 75 | DE | Scotland |
| 5 | 5 | Inner City private housing development. Quite friendly people. Under threat from rising sea levels. | Male | 63 | C1 | North East |
| 2 | 3 | Market Town | Male | 70 | AB | West Midlands |
| 3 | 3 | Quiet. Friendly & very pleasant | Male | 85 | C1 | West Midlands |
| 3 | 3 | It's OK | Female | 42 | AB | West Midlands |
| 3 | 4 | A small village | Female | 56 | DE | North East |
| 4 | 3 | Continual building destroying openness | Female | 66 | C1 | Greater London |
| 4 | 3 | I love that is very green | Female | 43 | C1 | Greater London |
| 3 | 3 | I feel very lucky to have green fields within five minutes from my house | Female | 73 | C1 | North West |
| 3 | 3 | Home | Female | 24 | C2 | The South East |
| 2 | 2 | The perfect mix. | Male | 36 | C1 | The South East |
| 2 | 3 | Lots of green fields and lovely village | Female | 70 | C1 | Greater London |
| 5 | 4 | A commuy | Male | 54 | DE | Greater London |
| 5 | 3 | Lifestyle | Male | 29 | AB | Greater London |
| 5 | 1 | It's my hometown | Male | 34 | C1 | East Midlands |
| 4 | 3 | Friendly | Male | 25 | AB | Greater London |
| 3 | 3 | Nice village/small town location with lovely countryside on our doorstep. | Male | 72 | C2 | Greater London |
| 5 | 5 | Roots | Male | 26 | AB | The South East |
| 1 | 1 | Very quiet and nice to live in the country | Male | 70 | C2 | The South West |
| 4 | 4 | Nothing its boring | Male | 42 | DE | Scotland |
| 3 | 5 | Peace | Female | 62 | DE | East of England |
| 4 | 3 | Convenient base | Female | 50 | C1 | Scotland |
| 4 | 1 | Not much I’d rather live in the country | Male | 20 | AB | West Midlands |
| 4 | 3 | Not much | Female | 28 | AB | Greater London |
| 3 | 4 | Community | Male | 34 | C2 | The South East |
| 2 | 1 | It’s my home | Female | 52 | AB | East of England |
| 3 | 1 | Too many immigrants | Female | 71 | DE | North West |
| 3 | 3 | Green spaces taken over by house building | Female | 37 | AB | Scotland |
| 5 | 3 | Means an awful lot | Female | 24 | C1 | East of England |
| 5 | 4 | Lively, fun, busy | Female | 43 | AB | Scotland |
| 3 | 4 | I love it | Female | 21 | C2 | East Midlands |
| 4 | 3 | nothing | Female | 65 | C1 | Greater London |
| 3 | 5 | Close to amenities and nature, close to sea level and susceptible to future flooding | Male | 57 | C1 | The South West |
| 1 | 1 | Gorgeous and peaceful | Male | 57 | C2 | East of England |
| 4 | 3 | I don't understand what this question is asking | Female | 44 | AB | West Midlands |
| 3 | 5 | There is always a lot of rubbish thrown down, including face masks. There is a steelworks about 5 miles from where I live, which creates lots of pollution into the air. There are many cases of Asthma in town and surrounding area, due to the pollution. | Female | 59 | C1 | East Midlands |
| 3 | 4 | I love where I live on the coast. Beautiful beaches and outdoor spaces to enjoy. | Female | 26 | C1 | Scotland |
| 4 | 3 | Community | Female | 46 | C2 | West Midlands |
| 3 | 3 | Friendly community, tidy area, local lakes and shops | Female | 47 | C1 | West Midlands |
| 5 | 4 | Home | Female | 43 | C2 | North West |
| 4 | 5 | Somewhere to live and work | Female | 59 | C2 | Greater London |
| 3 | 2 | On the decline | Female | 38 | C2 | North East |
| 2 | 1 | friendly | Female | 61 | DE | The South East |
| 3 | 5 | Tranquil | Female | 64 | DE | The South West |
| 3 | 2 | Typical housing estate | Male | 46 | DE | Scotland |
| 2 | 4 | Everything | Female | 31 | AB | West Midlands |
| 2 | 3 | Comfort | Male | 24 | DE | East Midlands |
| 3 | 5 | no | Female | 48 | DE | The South West |
| 4 | 3 | Ran down | Female | 24 | C1 | East of England |
| 3 | 3 | it was great but is now overdeveloped | Female | 72 | DE | The South West |
| 2 | 2 | Place to live nothing special | Male | 37 | AB | The South West |
| 4 | 3 | Friendly | Female | 33 | C1 | East Midlands |
| 3 | 3 | small town | Female | 65 | C2 | Scotland |
| 5 | 3 | Want to look after it for further generations | Female | 49 | DE | North West |
| 3 | 2 | Ok | Male | 29 | C1 | North West |
| 5 | 4 | Scary | Male | 45 | C2 | Greater London |
| 1 | 3 | Quite | Female | 73 | C2 | Scotland |
| 5 | 2 | London | Female | 42 | DE | Greater London |
| 3 | 3 | It's where I live | Male | 52 | C2 | North West |
| 2 | 5 | Tranquility | Male | 68 | C1 | North West |
| 3 | 4 | It doesn’t mean much to me | Female | 20 | C2 | North East |
| 5 | 3 | Great | Male | 69 | C1 | Scotland |
| 2 | 5 | IT HAS A GREAT HERITAGE AND WAS A REALLY GREEN AREA NOW TO MUCH HAS BEEN BUILT AND WE HAVE GONE FROM A VILLAGE TO A MINI TOWN | Male | 72 | C2 | Greater London |
| 3 | 2 | Home | Female | 57 | DE | East of England |
| 3 | 1 | Nothing | Female | 30 | C2 | Scotland |
| 5 | 3 | Just a place to live | Male | 70 | AB | Greater London |
| 3 | 3 | Home | Male | 23 | C1 | The South East |
| 5 | 3 | It's what I call home | Female | 51 | DE | The South East |
| 1 | 5 | Coastal area | Male | 76 | AB | North East |
| 3 | 3 | Good | Female | 27 | C1 | The South East |
| 1 | 5 | Home area | Female | 57 | C2 | East of England |
| 4 | 3 | It's run down | Female | 27 | AB | East Midlands |
| 3 | 1 | Local friendly | Male | 54 | C2 | Greater London |
| 4 | 1 | It's nice innit | Female | 21 | C2 | The South West |
| 2 | 2 | Beautiful countryside | Female | 68 | C2 | The South East |
| 4 | 2 | Comfortable | Male | 77 | DE | North East |
| 5 | 5 | Absolutely going down hill fast | Female | 72 | C2 | West Midlands |
| 3 | 2 | It’s nice and quite the neighbourhood is okay | Male | 80 | DE | The South West |
| 3 | 3 | Familiar | Female | 70 | AB | West Midlands |
| 3 | 3 | It's very safe, friendly and quiet. | Female | 24 | AB | The South East |
| 2 | 1 | lovely country views by river and lovely neighbours | Female | 54 | C1 | The South West |
| 2 | 5 | Normally quiet apart from English invasion every time the sun shines. | Male | 73 | C1 | Wales |
| 5 | 2 | Home | Male | 64 | DE | The South East |
| 5 | 3 | Home | Female | 41 | AB | Scotland |
| 3 | 4 | Friendly calm peaceful | Female | 56 | DE | East of England |
| 3 | 5 | Very pleasant | Male | 71 | C1 | North West |
| 4 | 3 | Could be improved | Female | 41 | C1 | Greater London |
| 3 | 3 | Nice to be there | Male | 44 | AB | East Midlands |
| 2 | 3 | my home | Female | 41 | AB | North West |
| 4 | 5 | Community | Male | 61 | C1 | North West |
| 2 | 2 | Sea is only 1-2 miles away, and 2 rivers plenty flow through the outskirts of the Town. There is also plenty of greens areas and parks. | Male | 72 | C1 | Scotland |
| 5 | 2 | easy to get around | Male | 50 | DE | Greater London |
| 2 | 3 | Home is where the heart is | Female | 49 | C2 | North East |
| 5 | 3 | Beautiful- near the sea & New forest....plus a choice of shops & work on my doorstep | Female | 49 | C1 | The South West |
| 2 | 3 | Town | Male | 50 | C2 | North East |
| 4 | 3 | It's my home. For now. | Female | 44 | C1 | The South East |
| 5 | 4 | it's where i live | Male | 32 | AB | Greater London |
| 3 | 2 | Peaceful | Female | 44 | AB | The South West |
| 2 | 3 | Friendly | Female | 49 | AB | East of England |
| 5 | 1 | Easy paced seaside town | Male | 69 | C1 | The South West |
| 3 | 4 | Nothing after all the buildings going up on green areas. | Male | 49 | C2 | The South East |
| 3 | 4 | The place where I live. | Female | 57 | DE | Scotland |
| 4 | 3 | It’s lovely and residential and I would love to carry on living here in the future sometime after I leave after university. | Female | 21 | AB | Greater London |
| 2 | 3 | worryingly the amount of new property being added to the green belt which is 99% flood plain so the localised flooding can only get worse | Male | 52 | C1 | North East |
| 2 | 3 | Open | Male | 33 | AB | North West |
| 3 | 4 | No | Female | 44 | C1 | North East |
| 2 | 1 | Reasonably quiet but a bit remote from shops etc. | Female | 71 | C2 | The South West |
| 4 | 5 | A lovely quiet area with friendly neighbours. But with always the worry as it is on a flood plain protected by sea defences and a river that has caused flooding before, but has had work done which has stopped flooding for years now. | Male | 52 | DE | Wales |
| 2 | 2 | Home | Male | 52 | DE | Scotland |
| 3 | 1 | Home | Male | 57 | C2 | East Midlands |
| 1 | 3 | Farming community would be in trouble | Male | 59 | DE | West Midlands |
| 4 | 4 | Pollution from landfill and motorways offset with fields and trees. | Female | 34 | C1 | Scotland |
| 2 | 4 | Horses | Female | 38 | AB | The South East |
| 3 | 3 | Friendly | Male | 70 | AB | West Midlands |
| 5 | 5 | Home | Male | 47 | AB | East of England |
| 3 | 4 | It’s home and that’s all that matters to me | Male | 40 | C1 | North East |
| 3 | 2 | Great views | Female | 52 | C1 | North East |
| 4 | 2 | Quiet suburbs | Male | 27 | DE | East of England |
| 3 | 3 | Lovely area | Female | 47 | C2 | East Midlands |
| 4 | 5 | home | Female | 36 | AB | East of England |
| 4 | 3 | Costal | Male | 76 | C1 | Scotland |
| 3 | 4 | It's home | Male | 41 | AB | East of England |
| 2 | 1 | Valley | Male | 52 | DE | Wales |
| 3 | 4 | Suburban | Female | 39 | C1 | East Midlands |
| 5 | 4 | Poo | Male | 37 | C2 | The South West |
| 2 | 3 | Home | Female | 23 | DE | East Midlands |
| 5 | 5 | Home | Male | 33 | AB | The South East |
| 3 | 2 | Village | Female | 33 | C1 | North East |
| 2 | 5 | Home | Female | 28 | DE | North West |
| 3 | 2 | Picturesque | Male | 42 | AB | North East |
| 3 | 3 | Home | Female | 50 | C1 | North West |
| 3 | 3 | good place to live | Male | 75 | DE | North East |
| 4 | 5 | Inclusive to all. | Male | 32 | C1 | The South East |
| 4 | 2 | We live in the outskirts of town but plenty of places to walk, we are 10 minutes from countryside. | Female | 56 | DE | North West |
| 4 | 4 | Leafy suburb | Male | 29 | C1 | North East |
| 2 | 2 | Rural and near the sea - where I live is my "heaven on earth" | Female | 87 | DE | The South East |
| 5 | 2 | Hom4 | Male | 80 | C2 | Wales |
| 3 | 5 | ITS MY HOME | Male | 54 | DE | North West |
| 5 | 1 | Home | Female | 63 | DE | North West |
| 3 | 1 | Zero | Male | 62 | DE | North West |
| 4 | 3 | Meh | Male | 35 | C2 | East Midlands |
| 3 | 4 | good mix of town and country | Male | 27 | AB | The South East |
| 2 | 5 | Home | Female | 22 | DE | East of England |
| 4 | 3 | home | Female | 35 | C2 | Greater London |
| 5 | 1 | I’m in a city | Female | 40 | DE | The South East |
| 3 | 3 | Ideal | Male | 53 | DE | Greater London |
| 3 | 4 | It’s a place to live | Female | 24 | C2 | The South East |
| 1 | 3 | Not a nice place | Male | 72 | DE | Greater London |
| 3 | 4 | dont know | Male | 46 | DE | The South West |
| 5 | 4 | Newcastle | Male | 56 | C2 | North East |
| 5 | 3 | Nice | Female | 51 | AB | North East |
| 3 | 2 | Suburbia in the north east. Hate it here, want to move somewhere away from people | Male | 31 | AB | North East |
| 5 | 3 | Suitable | Female | 20 | DE | West Midlands |
| 4 | 3 | No | Female | 33 | C1 | North East |
| 3 | 3 | Noce | Male | 32 | AB | Greater London |
| 3 | 5 | On the seafront | Female | 24 | C2 | Scotland |
| 3 | 4 | Peaceful, friendly, fairly green. | Female | 66 | AB | Scotland |
| 5 | 3 | Safe to bring my kids up | Female | 28 | DE | North East |
| 5 | 4 | Home | Female | 28 | C1 | Greater London |
| 5 | 2 | Good | Male | 64 | DE | Greater London |
| 3 | 1 | A perfect mix of rural and city | Male | 38 | AB | North East |
| 4 | 4 | I have lived here for half my life and am very attached to the area. | Male | 67 | C1 | East Midlands |
| 4 | 4 | Stop gap | Male | 44 | AB | Greater London |
| 2 | 2 | Forest | Female | 41 | DE | The South West |
| 2 | 2 | It’s peaceful when the tourists leave us alone | Female | 60 | AB | North East |
| 5 | 1 | Just a city | Male | 34 | AB | West Midlands |
| 4 | 4 | Glasgow | Female | 22 | C1 | Scotland |
| 1 | 4 | Beautiful views over the countryside and nearly on the coast. | Male | 69 | C2 | The South West |
| 1 | 1 | Saf | Female | 30 | C2 | The South East |
| 4 | 4 | it's a town suberb | Male | 66 | C1 | North West |
| 4 | 4 | Live quite close to Green Belt which is being built on & bought up for out if town centre living spaces.  Not even homes for families, just flats & apartments for wealthy commuters. | Male | 52 | DE | North West |
| 5 | 4 | The cycle lanes are causing more pollution as there are attempts to change things during a pandemic and TFL are not thinking at all. | Female | 41 | AB | Greater London |
| 3 | 2 | happy home | Male | 69 | C2 | North East |
| 3 | 5 | A lot | Male | 20 | C2 | The South East |
| 3 | 1 | I live in England | Female | 39 | C2 | North East |
| 3 | 2 | Very mixed, some nice parts, some awful parts | Male | 69 | C1 | Greater London |
| 3 | 3 | Town, assessable, friendly | Female | 33 | AB | West Midlands |
| 5 | 4 | It's home. | Male | 37 | C1 | North East |
| 3 | 1 | home | Male | 73 | AB | North East |
| 4 | 4 | It means the world to me as it is my home and im currently working as part of a big group to save a local green area which is the last green area vlosest to me from being taken over and turned into rubber 3g pitches and a massive car park | Female | 29 | AB | North West |
| 4 | 3 | It's my home | Male | 40 | C2 | North East |
| 5 | 4 | Famikt | Male | 19 | AB | Greater London |
| 1 | 4 | Home | Female | 30 | C2 | The South West |
| 5 | 4 | Good people, near to the sea beautiful scenery not too far away. | Female | 69 | AB | North East |
| 3 | 5 | Love it | Female | 37 | DE | North West |
| 2 | 5 | Small town was very quiet and peaceful, lovely place to live and raise a family and now to live a nice retirement life. I love it. | Male | 73 | AB | East of England |
| 5 | 2 | Quiet green area | Female | 43 | DE | Greater London |
| 4 | 3 | A large town | Male | 54 | C1 | West Midlands |
| 3 | 2 | Home | Male | 24 | AB | The South East |
| 1 | 4 | beautiful, quiet, single track, very small village looking out to a bay on west coast and selfishly don't want too many people here. | Male | 57 | C1 | Scotland |
| 4 | 4 | Lovely views | Female | 51 | C2 | North West |
| 4 | 3 | Green space means a lot to me, especially living on a city | Female | 36 | AB | North East |
| 3 | 5 | Everything | Male | 38 | C1 | The South West |
| 3 | 3 | Changing | Female | 24 | AB | The South East |
| 2 | 4 | Fresh air open fields 5 mins walk away | Female | 58 | C2 | North West |
| 3 | 3 | I’ve lived here all my childhood, it’s getting more built up as time goes on. | Female | 27 | AB | West Midlands |
| 5 | 2 | Born here, moved away 30 miles, moved to the USA for 7 years then moved right back here. Full circle. It's Home! My next door neighbour is my secondary school too. | Female | 65 | DE | North West |
| 4 | 3 | Nice to have green areas to walk the dog | Male | 30 | C1 | West Midlands |
| 5 | 4 | Crap | Male | 62 | C2 | North East |
| 4 | 5 | Everything at my doorsteps... | Female | 44 | DE | North West |
| 3 | 3 | Love it. Green spaces right outside | Female | 45 | C1 | Scotland |
| 5 | 3 | Suburban | Male | 74 | AB | Greater London |
| 3 | 2 | Home | Male | 39 | C1 | Greater London |
| 3 | 4 | gridlocked traffic, lack of dentist, need new hospital | Male | 50 | DE | East of England |
| 5 | 1 | somewhere to live | Male | 65 | DE | The South East |
| 5 | 5 | Council estate neglected by council | Female | 64 | DE | North West |
| 2 | 3 | Nice | Female | 32 | C2 | Greater London |
| 2 | 1 | pleasant little market town with friendly people | Female | 77 | C1 | North West |
| 3 | 2 | not from area | Male | 34 | AB | Greater London |
| 5 | 4 | Love it | Male | 31 | C2 | The South West |
| 1 | 3 | Yes | Female | 35 | AB | Wales |
| 3 | 2 | A place to live | Female | 25 | AB | The South East |
| 5 | 5 | Simply where I live | Male | 37 | AB | Greater London |
| 2 | 3 | Village life and community | Female | 67 | AB | North East |
| 4 | 5 | Where I’ve grown up | Male | 28 | C1 | Scotland |
| 3 | 2 | Home | Male | 23 | DE | West Midlands |
| 3 | 4 | Community | Male | 42 | C2 | North West |
| 4 | 4 | Everything is in close proximity | Female | 36 | C2 | West Midlands |
| 5 | 3 | Violent | Male | 49 | AB | Greater London |
| 3 | 5 | Home, countryside and hireath | Female | 22 | AB | Wales |
| 2 | 4 | Beautiful countryside with beach close by | Male | 33 | C2 | The South West |
| 2 | 2 | Quiet farming village | Female | 39 | DE | The South West |
| 3 | 5 | Not much | Male | 36 | C1 | West Midlands |
| 5 | 5 | I love my local area. There are plenty of shops so you can get everything you need within walking distance of home. There is also alot of parkland not too far away. We also have the best two football teams Everton and Liverpool in our city. Some crime but it is everywhere anyway. | Female | 64 | DE | North West |
| 3 | 2 | Very nice, greenery bits would like more | Female | 28 | C2 | West Midlands |
| 2 | 2 | Quiet | Male | 41 | AB | East Midlands |
| 3 | 2 | It's where I live | Female | 52 | DE | North West |
| 2 | 2 | Home | Female | 41 | C1 | North West |
| 2 | 2 | Countryside, peaceful. | Female | 23 | DE | North West |
| 4 | 3 | Meh | Male | 38 | DE | The South East |
| 2 | 2 | Most famous village in the country, a beautiful large village with lots of history. | Female | 28 | AB | West Midlands |
| 2 | 2 | Home | Male | 59 | DE | East of England |
| 3 | 4 | Ok | Female | 33 | AB | East of England |
| 2 | 5 | Great | Male | 51 | DE | The South East |
| 3 | 2 | Peace and quiet | Male | 80 | C2 | Wales |
| 4 | 1 | Quiet and out of the way | Male | 120 | DE | North West |
| 3 | 3 | Home | Female | 28 | C2 | East Midlands |
| 4 | 3 | No | Female | 64 | DE | North West |
| 5 | 2 | Dump | Male | 34 | C2 | North West |
| 4 | 2 | home | Female | 21 | AB | Wales |
| 3 | 4 | Flooding | Female | 24 | AB | North West |
| 3 | 3 | Dirty steel works town full of lovely people. | Male | 67 | C1 | Wales |
| 1 | 1 | Bliss | Male | 53 | DE | West Midlands |
| 3 | 3 | Quite relaxing. Lots of nature | Male | 38 | C1 | North East |
| 4 | 3 | It’s a nice area | Male | 52 | C1 | The South East |
| 4 | 4 | Little village, very friendly, welcoming | Female | 22 | C1 | East Midlands |
| 1 | 3 | peace and quiet. | Male | 70 | C1 | Scotland |
| 2 | 3 | Safe | Female | 43 | C1 | West Midlands |
| 3 | 3 | Home | Female | 23 | AB | East Midlands |
| 4 | 3 | Home | Male | 26 | DE | West Midlands |
| 4 | 3 | Too much new build without enough facilities | Male | 61 | C2 | Greater London |
| 3 | 3 | None | Female | 70 | C1 | East Midlands |
| 4 | 3 | Lost open space | Male | 52 | C1 | Scotland |
| 3 | 3 | Nowt cause I can’t stand it, all green spaces are either covered or going to be covered with houses. | Female | 19 | DE | North East |
| 3 | 3 | Town | Female | 59 | C2 | Scotland |
| 3 | 3 | Township | Male | 51 | C2 | West Midlands |
| 3 | 3 | Home | Male | 27 | C1 | The South East |
| 2 | 4 | Nothing | Female | 40 | AB | North East |
| 2 | 3 | Quaint, well-connected | Female | 25 | AB | East of England |
| 2 | 2 | semi-rural countryside yet near town | Female | 71 | AB | East Midlands |
| 2 | 5 | Loss of green space and trees | Female | 65 | AB | Greater London |
| 4 | 1 | Quite high density housing but lots of green space | Female | 66 | C1 | The South East |
| 2 | 5 | Rural, green and quiet | Male | 34 | AB | East Midlands |
| 2 | 2 | Home | Male | 35 | DE | North West |
| 3 | 2 | Lovely quiet green estate | Male | 36 | C1 | East Midlands |
| 5 | 3 | London | Male | 32 | C1 | Greater London |
| 3 | 4 | Safe from muggers | Male | 62 | AB | Greater London |
| 4 | 4 | Nothing | Female | 39 | DE | The South East |
| 2 | 4 | Home | Male | 55 | DE | The South West |
| 3 | 3 | Town | Female | 30 | DE | The South West |
| 3 | 5 | Hate it | Male | 33 | C1 | The South East |
| 3 | 4 | Near the sea. Good place to live. | Male | 58 | DE | East of England |
| 2 | 4 | Quiet small town | Female | 68 | DE | The South East |
| 3 | 3 | Terrible | Male | 23 | DE | East Midlands |
| 4 | 5 | Crap hole | Male | 41 | AB | Greater London |
| 2 | 3 | Home | Male | 38 | C1 | East Midlands |
| 4 | 5 | no | Male | 33 | AB | Greater London |
| 2 | 5 | sea side | Female | 65 | AB | The South East |
| 5 | 3 | Too many houses built with not enough jobs, Doctors, and schools to accommodate | Female | 57 | C2 | The South West |
| 4 | 1 | Quiet mostly | Female | 61 | DE | East Midlands |
| 2 | 3 | Nice | Male | 49 | AB | West Midlands |
| 2 | 3 | Born and bred in this area, it's very important to me and my family. | Male | 64 | C2 | The South West |
| 5 | 3 | A quiet area where I feel safe, with lots of clean parks and green spaces, but too many cars in front of houses and pollution. | Female | 40 | C2 | Greater London |
| 4 | 3 | Ok | Female | 73 | AB | East of England |
| 1 | 3 | Changing for the better | Female | 47 | AB | The South West |
| 3 | 5 | destruction of the planet | Male | 46 | C1 | The South West |
| 4 | 1 | friendly and peaceful | Female | 58 | DE | The South West |
| 4 | 3 | Like it | Female | 58 | C2 | Scotland |
| 4 | 4 | pleasant, quiet, safe | Male | 39 | C2 | West Midlands |
| 3 | 3 | Nice village | Female | 44 | C1 | The South East |
| 4 | 4 | Could be more pretty needs some regeneration | Female | 53 | C1 | Greater London |
| 3 | 3 | Countryside but still able to get to residential area easily | Female | 53 | C1 | East of England |
| 3 | 3 | Cool, calm and collected (at the moment ) | Male | 83 | C1 | North East |
| 5 | 2 | Just a place to live | Male | 64 | DE | Greater London |
| 2 | 4 | Nive | Female | 21 | C1 | East of England |
| 3 | 4 | Nature | Female | 21 | DE | Wales |
| 3 | 3 | It is cool | Male | 33 | C1 | North East |
| 2 | 3 | I love living in my neighbour | Female | 51 | C2 | North East |
| 4 | 3 | Beautiful | Female | 53 | C1 | Scotland |
| 3 | 4 | A lovely area for residing and recreation | Male | 84 | DE | North East |
| 4 | 3 | Nice and mostly quiet | Male | 68 | C1 | The South East |
| 3 | 3 | Home | Female | 41 | C1 | East Midlands |
| 3 | 3 | It is my home | Male | 68 | DE | East of England |
| 5 | 4 | Community | Female | 65 | AB | North East |
| 3 | 3 | Great | Female | 22 | DE | Greater London |
| 2 | 4 | I don't know | Male | 24 | DE | The South West |
