# Supplementary material for: Place attachment and perception of climate change as a threat in rural and urban areas
Source: PLoS One. 2023 Sep 6;18(9):e0290354. doi: 10.1371/journal.pone.0290354 (PMC10482299; doi:10.1371/journal.pone.0290354)
Supplement: S2 Table — This is visually represented in S1 Fig. Note, rurality is an ordinal variable and so the model fits a series of polynomial functions to the levels of the variable: the first is linear (.L), the second is quadratic (.Q), the third is cubic (.C), and the last (^4) is to the power four. (DOCX) [file pone.0290354.s002.docx]

**S2 Table. The output of the statistical model represented in Equation 2.** This is visually represented in Fig SI-1. Note, rurality is an ordinal variable and so the model fits a series of polynomial functions to the levels of the variable: the first is linear (.L), the second is quadratic (.Q), the third is cubic (.C), and the last (^4) is to the power four.

| **Term** | **Value** | **Std. Error** | **t value** | **p value** |
| --- | --- | --- | --- | --- |
| Rurality.L | 1.227846038 | 0.268616372 | 4.571002235 | 4.85E-06 |
| Rurality.Q | -0.502440213 | 0.230961003 | -2.175433107 | 0.029597676 |
| Rurality.C | 0.330537834 | 0.194197817 | 1.70206771 | 0.088742676 |
| Rurality^4 | -0.226613859 | 0.146195267 | -1.55007658 | 0.121123136 |
| GenderMale | -0.222303607 | 0.151092793 | -1.471305159 | 0.141208609 |
| SEGC1 | 0.162727794 | 0.198611449 | 0.819327359 | 0.412599667 |
| SEGC2 | -0.076466925 | 0.214656933 | -0.356228535 | 0.721669424 |
| SEGDE | -0.118440078 | 0.208861927 | -0.567073568 | 0.5706642 |
| RegionEast of England | 0.43091009 | 0.349105501 | 1.234326266 | 0.217081352 |
| RegionGreater London | 0.287543696 | 0.330678999 | 0.86955536 | 0.384543442 |
| RegionNorth East | -0.022619374 | 0.315057925 | -0.071794336 | 0.94276558 |
| RegionNorth West | 0.273266695 | 0.344559313 | 0.793090435 | 0.427725135 |
| RegionScotland | 0.353815658 | 0.365110247 | 0.969065264 | 0.332512627 |
| RegionThe South East | 0.593352751 | 0.321789449 | 1.843916114 | 0.065195366 |
| RegionThe South West | 0.618420879 | 0.351862085 | 1.757566116 | 0.078821363 |
| RegionWales | 0.282217181 | 0.40453471 | 0.697634031 | 0.485406093 |
| RegionWest Midlands | 0.66269413 | 0.336982281 | 1.966554821 | 0.049234556 |
| Age_cat30-39 | -0.247717439 | 0.245382902 | -1.009513856 | 0.31272826 |
| Age_cat40-49 | -0.27804285 | 0.251867439 | -1.103925345 | 0.269625523 |
| Age_cat50-59 | -0.518916508 | 0.255575745 | -2.030382452 | 0.042317679 |
| Age_cat60-69 | -0.455184526 | 0.253663711 | -1.794440848 | 0.072742832 |
| Age_cat70-79 | -0.932392261 | 0.323835271 | -2.879217752 | 0.00398663 |
| Age_cat80+ | -1.359676694 | 0.546628246 | -2.487388282 | 0.012868486 |
| Place.Attachment.Score | -0.044579554 | 0.037933236 | -1.175210944 | 0.239910333 |
| wordcount | 0.028992475 | 0.011894753 | 2.43741706 | 0.014792607 |
| 1 - not a threat at all\|2 | -2.27023289 | 0.351152687 | -6.46508763 | 1.01E-10 |
| 2\|3 | -1.003269656 | 0.337359648 | -2.973887549 | 0.002940528 |
| 3\|4 | 0.8347148 | 0.337473165 | 2.473425703 | 0.013382463 |
| 4\|5 - extremely threatening | 2.103473469 | 0.347238529 | 6.057719106 | 1.38E-09 |
