# Supplementary material for: Place attachment and perception of climate change as a threat in rural and urban areas
Source: PLoS One. 2023 Sep 6;18(9):e0290354. doi: 10.1371/journal.pone.0290354 (PMC10482299; doi:10.1371/journal.pone.0290354)
Supplement: S3 Table — Note, rurality is an ordinal variable and so the model fits a series of polynomial functions to the levels of the variable: the first is linear (.L), the second is quadratic (.Q), the third is cubic (.C), and the last (^4) is to the power four. (DOCX) [file pone.0290354.s003.docx]

**S3 Table. The output of the statistical model represented in Equation 3.** Note, rurality is an ordinal variable and so the model fits a series of polynomial functions to the levels of the variable: the first is linear (.L), the second is quadratic (.Q), the third is cubic (.C), and the last (^4) is to the power four.

| **term** | **estimate** | **std.error** | **statistic** | **p.value** |
| --- | --- | --- | --- | --- |
| (Intercept) | 1.587512586 | 0.282391172 | 5.621679231 | 2.43E-08 |
| Rurality.L | -0.575693876 | 0.201115265 | -2.862507113 | 0.004286875 |
| Rurality.Q | -0.18762026 | 0.172363823 | -1.088512986 | 0.276619437 |
| Rurality.C | 0.092960592 | 0.151423466 | 0.613911401 | 0.539407363 |
| Rurality^4 | 0.034111104 | 0.120935316 | 0.282060735 | 0.777952737 |
| GenderMale | -0.09494011 | 0.123482764 | -0.768853129 | 0.442154065 |
| SEGC1 | 0.186895227 | 0.164182418 | 1.138338861 | 0.255239666 |
| SEGC2 | 0.052629234 | 0.17958177 | 0.293065571 | 0.769530161 |
| SEGDE | -0.330221373 | 0.171412539 | -1.926471513 | 0.054316223 |
| RegionEast of England | 0.187430791 | 0.296749108 | 0.631613661 | 0.527777363 |
| RegionGreater London | -0.272046402 | 0.282894394 | -0.961653561 | 0.336445903 |
| RegionNorth East | 0.106486996 | 0.276446522 | 0.38519926 | 0.700168106 |
| RegionNorth West | 0.223497929 | 0.284436546 | 0.785756726 | 0.432187975 |
| RegionScotland | 0.371022581 | 0.301147451 | 1.232029625 | 0.218214896 |
| RegionThe South East | -0.065266848 | 0.273210255 | -0.238888721 | 0.811238667 |
| RegionThe South West | 0.279355773 | 0.299426045 | 0.932970853 | 0.35105032 |
| RegionWales | 0.473323186 | 0.346291843 | 1.366833194 | 0.171971288 |
| RegionWest Midlands | -0.215467914 | 0.29696949 | -0.725555728 | 0.468273464 |
| Age_cat30-39 | -0.304786305 | 0.201734618 | -1.510827973 | 0.131134309 |
| Age_cat40-49 | -0.003199376 | 0.203378575 | -0.015731138 | 0.987451885 |
| Age_cat50-59 | -0.203287947 | 0.215133494 | -0.944938619 | 0.344908431 |
| Age_cat60-69 | 0.09232167 | 0.208833521 | 0.44208262 | 0.658520807 |
| Age_cat70-79 | 0.115489192 | 0.247217151 | 0.467156877 | 0.640484939 |
| Age_cat80+ | -0.027558409 | 0.447150509 | -0.06163117 | 0.950868328 |
| wordcount | 0.148724383 | 0.008747184 | 17.00254462 | 1.98E-57 |

Residual standard error: 1.951 on 1046 degrees of freedom

Multiple R-squared: 0.266, Adjusted R-squared: 0.2492

F-statistic: 15.8 on 24 and 1046 DF, p-value: < 2.2e-16
