# Supplementary material for: Place attachment and perception of climate change as a threat in rural and urban areas
Source: PLoS One. 2023 Sep 6;18(9):e0290354. doi: 10.1371/journal.pone.0290354 (PMC10482299; doi:10.1371/journal.pone.0290354)
Supplement: S4 Table — This is visually represented in Fig 1. Note, rurality is an ordinal variable and so the model fits a series of polynomial functions to the levels of the variable: the first is linear (.L), the second is quadratic (.Q), the third is cubic (.C), and the last (^4) is to the power four. (DOCX) [file pone.0290354.s004.docx]

**S4 Table. The output of the statistical model represented in Equation 4.** This is visually represented in Figure 1. Note, rurality is an ordinal variable and so the model fits a series of polynomial functions to the levels of the variable: the first is linear (.L), the second is quadratic (.Q), the third is cubic (.C), and the last (^4) is to the power four.

| **Term** | **Value** | **Std. Error** | **t value** | **p value** |
| --- | --- | --- | --- | --- |
| Rurality.L | 1.297578993 | 0.256914937 | 5.05061717 | 4.40E-07 |
| Rurality.Q | -0.545366388 | 0.223270385 | -2.442627526 | 0.014580777 |
| Rurality.C | 0.361967139 | 0.187024945 | 1.935394975 | 0.052941846 |
| Rurality^4 | -0.320366885 | 0.143563558 | -2.231533466 | 0.025645813 |
| 1 - not a threat at all\|2 | -2.08773101 | 0.141370007 | -14.7678497 | 2.36E-49 |
| 2\|3 | -0.858123126 | 0.106273993 | -8.074629545 | 6.77E-16 |
| 3\|4 | 0.901376939 | 0.104848031 | 8.596984924 | 8.18E-18 |
| 4\|5 - extremely threatening | 2.136663377 | 0.133630483 | 15.98934108 | 1.52E-57 |
