# Supplementary material for: Place attachment and perception of climate change as a threat in rural and urban areas
Source: PLoS One. 2023 Sep 6;18(9):e0290354. doi: 10.1371/journal.pone.0290354 (PMC10482299; doi:10.1371/journal.pone.0290354)
Supplement: S5 Table — This is visually represented in Fig 2 and S2 Fig. Note, rurality is an ordinal variable and so the model fits a series of polynomial functions to the levels of the variable: the first is linear (.L), the second is quadratic (.Q), the third is cubic (.C), and the last (^4) is to the power four. (DOCX) [file pone.0290354.s005.docx]

**S5 Table. The output of the statistical model represented in Equation 5.** This is visually represented in Figure 2 and Figure SI-2. Note, rurality is an ordinal variable and so the model fits a series of polynomial functions to the levels of the variable: the first is linear (.L), the second is quadratic (.Q), the third is cubic (.C), and the last (^4) is to the power four.

| **term** | **estimate** | **std.error** | **statistic** | **p.value** |
| --- | --- | --- | --- | --- |
| (Intercept) | 1.526004974 | 0.083723133 | 18.22680218 | 8.12E-65 |
| Rurality.L | -0.715839779 | 0.192320553 | -3.722117922 | 0.000207892 |
| Rurality.Q | -0.170201371 | 0.170962496 | -0.995547997 | 0.319695948 |
| Rurality.C | 0.112471391 | 0.150941906 | 0.745130318 | 0.456357345 |
| Rurality^4 | 0.061423246 | 0.12050198 | 0.509728102 | 0.61034757 |
| wordcount | 0.152778405 | 0.00854515 | 17.87896209 | 1.01E-62 |

Residual standard error: 1.955 on 1065 degrees of freedom

Multiple R-squared: 0.2459, Adjusted R-squared: 0.2424

F-statistic: 69.47 on 5 and 1065 DF, p-value: < 2.2e-16
