# Supplementary material for: Place attachment and perception of climate change as a threat in rural and urban areas
Source: PLoS One. 2023 Sep 6;18(9):e0290354. doi: 10.1371/journal.pone.0290354 (PMC10482299; doi:10.1371/journal.pone.0290354)
Supplement: S1 Fig — Rurality was a significant predictor of perceived threat of climate change and is shown in Fig 1 (S2 Table). (DOCX) [file pone.0290354.s006.docx]

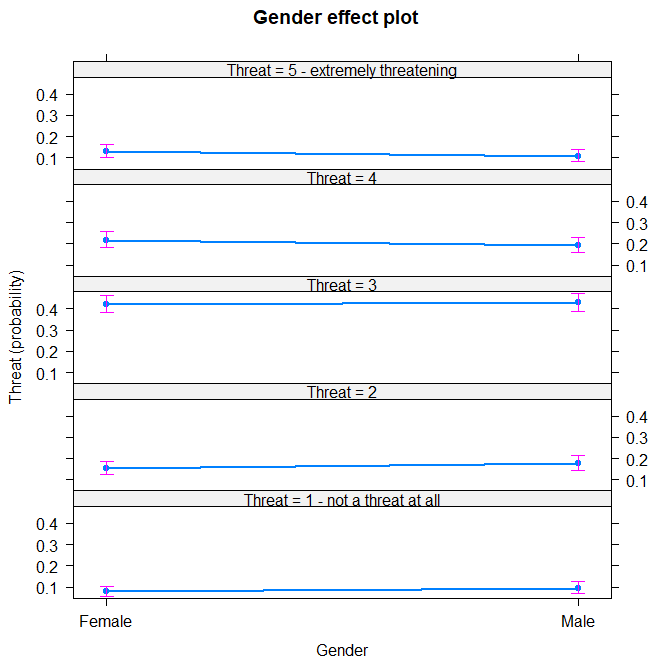

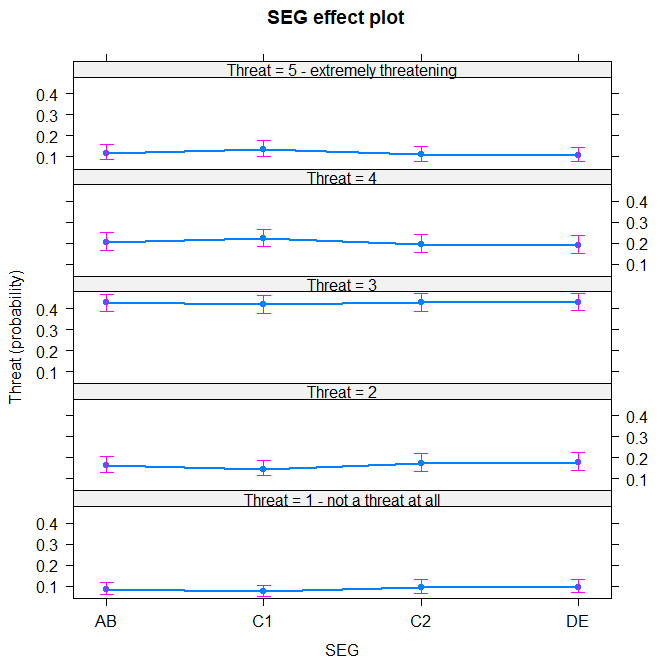

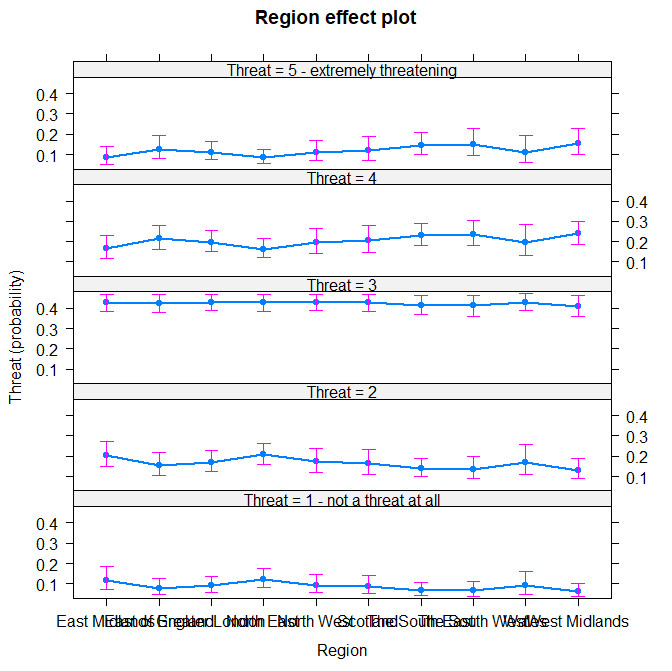

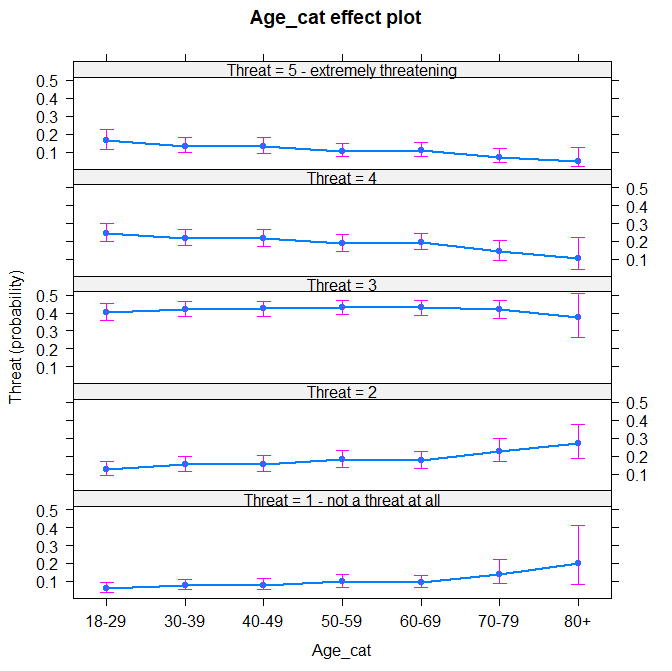

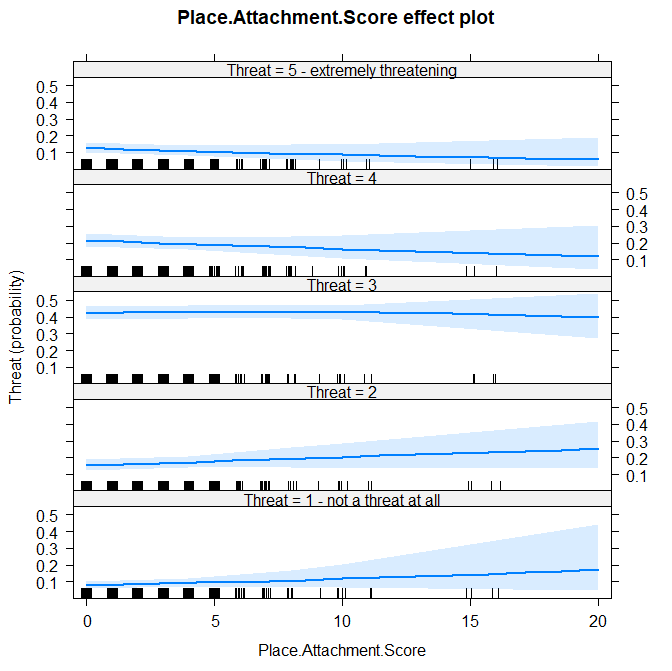

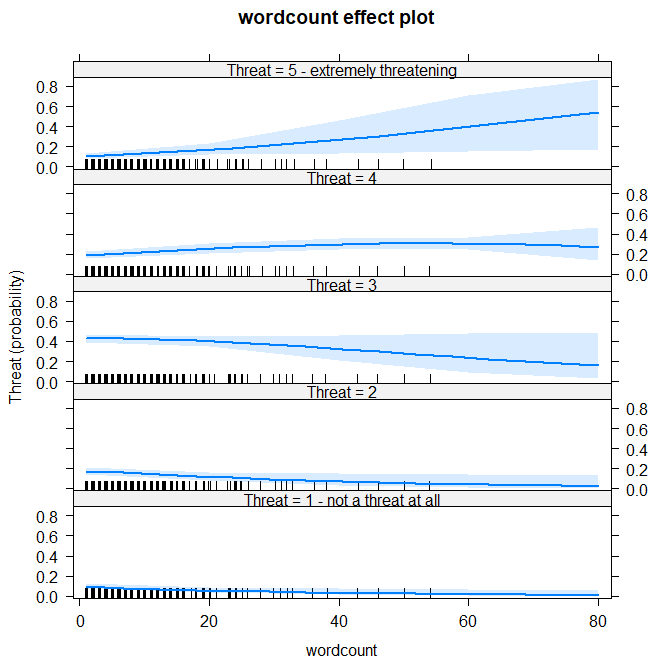


1. Socio-economic group
2. Gender
3. Wordcount
4. Place Attachment Score
5. Age
6. Region

**S1 Figure. An effects plot comparing explanatory variables with the perceived threat of climate change (n = 1,071; Eq. 1).** Rurality was a significant predictor of perceived threat of climate change and is shown in Figure 1 (S2 Table).
