# Supplementary material for: Place attachment and perception of climate change as a threat in rural and urban areas
Source: PLoS One. 2023 Sep 6;18(9):e0290354. doi: 10.1371/journal.pone.0290354 (PMC10482299; doi:10.1371/journal.pone.0290354)
Supplement: S2 Fig — Free text descriptions with higher word count obtained a higher place attachment score (with each additional word raising the score by 0.15; p < 0.001). Rurality was also a significant predictor of place attachment score, with the most rural people having the highest place scores (S3 Table). (DOCX) [file pone.0290354.s007.docx]

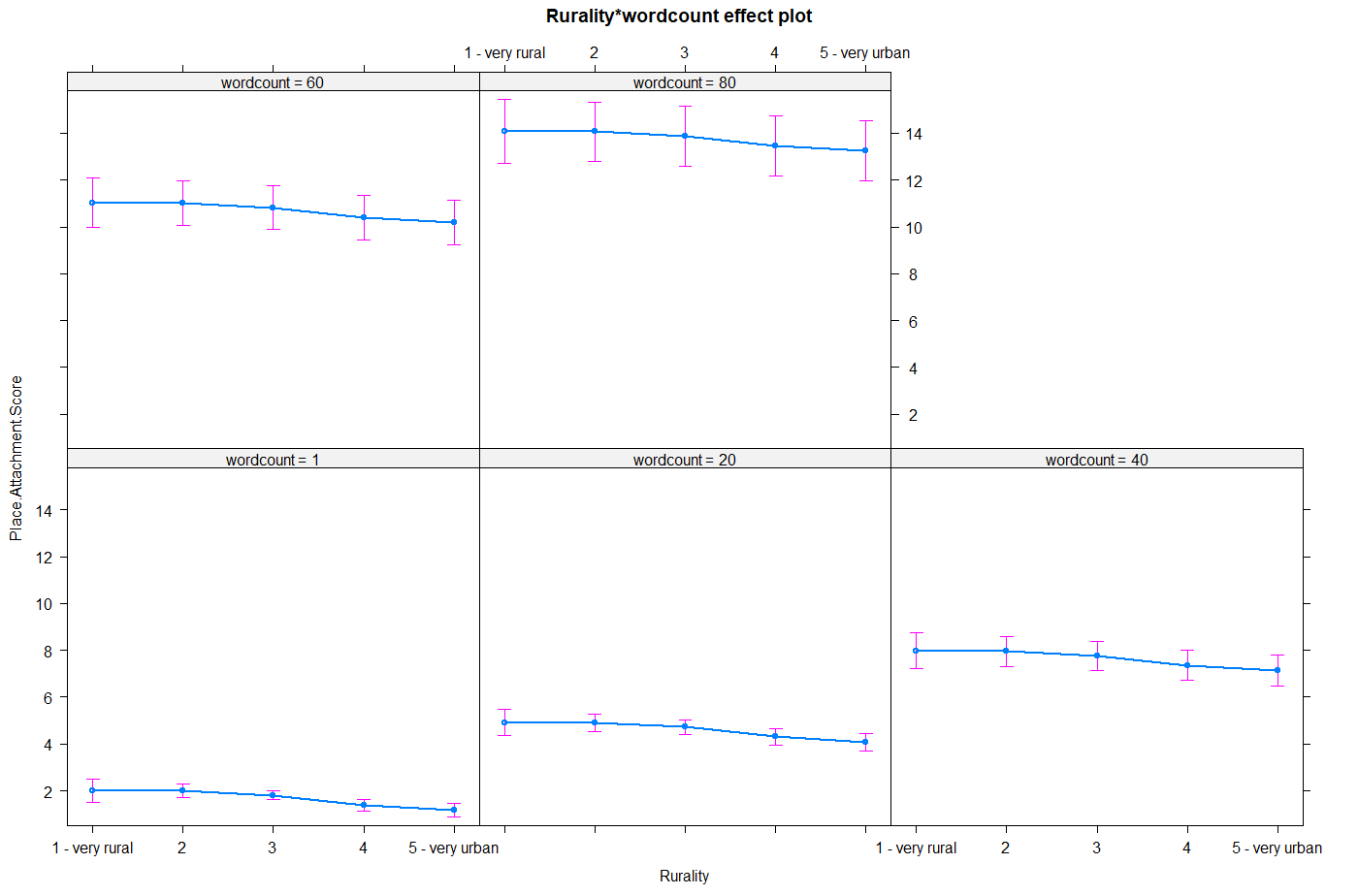


**S2 Figure. An effects plot comparing self-defined rurality and wordcount with the place attachment score (n = 1,071; Eq. 5).** Free text descriptions with higher word count obtained a higher place attachment score (with each additional word raising the score by 0.15; p < 0.001). Rurality was also a significant predictor of place attachment score, with the most rural people having the highest place scores (Table SI-3).
